# High-fidelity minute-level physiologic trajectories after ROSC from linked monitor-defibrillator recordings in out-of-hospital cardiac arrest

**DOI:** 10.1016/j.resplu.2026.101286

**Published:** 2026-03-06

**Authors:** Pieter Francsois Fouche, Emily Nehme, Sam Burton, Belinda Flanagan, Benjamin Meadley, David Anderson, Dion Stub, Ziad Nehme

**Affiliations:** a University of Tasmania, School of Paramedicine and Public Safety, Hobart, Australia; b Ambulance Victoria, Melbourne, Australia; c School of Public Health and Preventive Medicine, Monash University, Melbourne, Australia; d Department of Paramedicine, Monash University, Melbourne, Australia

**Keywords:** Cardiac arrest, Return of spontaneous circulation, Blood pressure, Oxygen, End-tidal carbon dioxide, Prehospital, Out-of-hospital

## Abstract

**Background:**

Early post-ROSC physiology changes rapidly, but most studies rely on a single handover set of vital signs, which can miss trajectories and time spent hypotensive or hypoxemic. We aimed to describe minute-level early post-ROSC blood pressure and oxygenation and examine their association with outcomes.

**Methods:**

Retrospective cohort study of out-of-hospital cardiac arrest in Victoria, Australia (2019–2023). We linked the Victorian Ambulance Cardiac Arrest Registry to Zoll® monitor-defibrillator recordings, aligned measurements to recorded ROSC, aggregated readings into 1-min bins, and derived per-patient mean, minimum, and minutes below thresholds for SBP, MAP and SpO_2_. Associations with outcomes were modelled using adjusted logistic regression. Primary outcome was survival to hospital discharge. Secondary outcome was good 12-month neurological outcome among survivors.

**Results:**

Among 3694 patients with sustained ROSC, 1444 survived. Median ROSC-to-arrival was 58 min. Median per patient was 36 blood pressure and 97 SpO_2_ values. Compared with SBP 100 mm Hg, survival was lower at 80 mm Hg (aOR 0.87, 95% CI 0.79–0.95) and higher at 140 mm Hg (aOR 1.32, 95% CI 1.11–1.59). SpO_2_ 90% versus 95% was associated with lower survival (aOR 0.70, 95% CI 0.63–0.79). Longer time with SBP <90 mm Hg, MAP <65 mm Hg, or SpO_2_ <90% was associated with lower survival. Associations with good 12-month neurological outcome were smaller and mainly limited to blood pressure.

**Conclusions:**

Minute-level monitor-defibrillator data showed strong associations between early hypotension and hypoxaemia and survival after ROSC and allow cumulative exposure to be quantified.

## Highlights

• Registry-linked, ROSC-aligned, minute-by-minute Zoll® monitor-defibrillator data.
• Enables cumulative exposure metrics for hypotension, hypoxaemia and ETCO_2_.
• Higher SBP/MAP and SpO_2_ were associated with greater survival to hospital discharge.
• Higher mean ETCO_2_ was inversely associated with survival.
• Neurological associations among survivors were smaller, mainly limited to SBP.

## Introduction

Out-of-hospital cardiac arrest (OHCA) is a major cause of death worldwide.^1^, ^2^ Current international guidelines recommend maintaining systolic blood pressure (SBP) ≥90 mm Hg, mean arterial pressure (MAP) ≥65 mm Hg, and avoiding hypoxaemia with oxygen saturation targeted to normoxaemic ranges once monitoring is reliable.^3^ However, it remains unclear how blood pressure and oxygenation levels in the immediate post-ROSC phase influence outcomes, particularly in the early prehospital window.

The immediate problem following return of spontaneous circulation (ROSC) is hemodynamic instability in the first minutes. Shock is frequently observed post-ROSC, with reported incidence in some cohorts ranging between 15% and 68%.^4^, ^5^, ^6^ Recent work has shown that not just the presence of hypotension, but its depth and cumulative duration are associated with poor survival.^7^ Together, these observations point to an early vulnerability window. However, randomised trials of post-ROSC blood pressure management, including BOX, have not shown benefit from higher MAP targets. Importantly, these trials did not test permissive hypotension in the immediate post-ROSC prehospital window, when hypotension is common, so they do not resolve whether early hypotension and cumulative exposure contribute to outcome.^8^ This mismatch between trial targets and observational signal suggests risk may accumulate across the continuous BP range. This accumulated risk is poorly understood because prior research is limited by collapsing systolic blood pressure into broad categories, thereby sacrificing data precision and obscuring important nonlinear relationships with survival.^9^

To address this gap, we analysed discrete prehospital cardiac and vital sign monitor data to characterise early post-ROSC blood pressure patterns and relate them to survival. The patient-level summaries derived from these recordings offer substantially higher resolution than earlier studies based on a single-point-in-time reading at hospital handover.

Oxygenation and ventilation are critical to recovery after cardiac arrest, as is management of hypoxic-ischaemic and other secondary brain injury processes, seizures, temperature and glucose, and the precipitating cause.^3^ The EXACT trial showed lower survival with restrictive oxygen targets, while TAME found no benefit from mild hypercapnia, leaving optimal oxygen and carbon dioxide control after ROSC uncertain.^10^, ^11^ Despite this uncertainty, contemporary guidelines recommend avoiding hypoxaemia, with oxygen titrated to a normoxaemic range once monitoring is reliable, commonly within 90–98%.^3^, ^12^ However, it remains unclear how blood pressure and oxygenation levels in the immediate post-ROSC phase influence outcomes, particularly in the early prehospital window. We aimed to describe minute-level post-ROSC trajectories and quantify how early blood pressure and oxygenation relate to survival and neurological outcome.

## Methods

This study is reported in accordance with the STROBE guidelines for cohort studies.^13^

### Study design and setting

Ambulance Victoria is the statewide EMS provider for a population of about 6.6 million, staffed by Advanced Life Support (ALS) paramedics and Mobile Intensive Care Ambulance (MICA) paramedics, with MICA authorised to perform endotracheal intubation and rapid sequence induction. Post-ROSC care is clinician-directed under statewide clinical practice guidelines and prioritises rapid transport while stabilising oxygenation, ventilation and perfusion. Oxygen is provided early and titrated once pulse oximetry is reliable. Ventilation is guided by capnography when available, using tidal volumes of 6–8 mL/kg and titrating ventilation to an ETCO_2_ target of 30–35 mmHg where feasible. Airway management is typically stepwise, using bag-valve-mask and/or a supraglottic airway (SGA) initially, with ETI used when required for airway protection or controlled ventilation, and may occur sequentially within the same case. Intravenous fluids are used judiciously for hypotension, with total fluid during arrest and post-ROSC management generally not exceeding 20 mL/kg unless correcting suspected hypovolaemia. If hypotension persists despite initial fluid (typically after 500–1000 mL with minimal improvement) or if profound hypotension is present (for example SBP <70 mmHg, altered mental status, or absent radial pulses), vasopressors may be administered, typically metaraminol boluses, and adrenaline or noradrenaline infusions delivered via electronic syringe drivers.

### Data sources

Registry records were linked to ambulance clinical records and Zoll X Series® monitor-defibrillator files. Time-stamped physiologic streams included systolic and diastolic blood pressure, mean arterial pressure, oxygen saturation, end-tidal carbon dioxide, and respiratory rate. Survival and neurological outcomes were ascertained via medical record review and structured 12-month follow-up by trained registry staff, using validated instruments, including the Glasgow Outcome Scale Extended (GOSE).

### Participants

We included adult OHCA patients with sustained ROSC, defined as a pulse on hospital arrival, with a retrievable linked monitor-defibrillator file and non-missing survival outcome data. Neurological analyses were restricted to survivors with 12-month GOSE available.

### Variables and definitions

Physiologic measures were aligned to the recorded time of return of spontaneous circulation and aggregated to 1-min intervals. For each patient we derived mean, minimum, and time-below-threshold measures for systolic blood pressure, mean arterial pressure, oxygen saturation, end-tidal carbon dioxide, and respiratory rate.

Prespecified covariates included age, sex, initial rhythm, downtime (call receipt to first sustained ROSC time recorded by paramedics), witnessed status, arrest location, presumed cause, and resuscitation drugs. Covariate selection was prespecified using a directed acyclic graph. Raw monitor measurements were recorded at irregular intervals and subsequently aggregated into 1-min bins, and handling of intermittent NIBP, plausibility screening, missingness, and minimum data requirements for cumulative exposure measures are detailed in the Supplementary Appendix.

Prespecified thresholds defined hypotension (SBP <90 mm Hg; MAP <65 mm Hg), hypoxaemia (SpO_2_ <90%), abnormal ETCO_2_ (<20 or >45 mm Hg), and abnormal respiratory rate (RR <8 or >30 breaths/min). ETCO_2_ and RR were obtained from the capnography channel. In ventilated patients, RR largely reflects delivered ventilation rather than spontaneous breathing, and both ETCO_2_ and RR are influenced by airway type, seal quality, ventilation strategy, pulmonary blood flow, and illness severity. Airway management and sedation are clinician-directed and may change over time, and ventilation mode or drug effect could not be reliably classified minute-by-minute, so these measures are interpreted as context-dependent markers rather than direct treatment targets.

The primary outcome was survival to hospital discharge. The secondary outcome was 12-month neurological status on GOSE, dichotomised as good (scores 7–8) versus not good (scores 1–6), aligning with the scientific statement on post cardiac arrest syndrome that recognises brain injury as the main cause of morbidity and mortality after ROSC.^14^

### Statistical analysis

Zoll® monitor data processing and quality assurance methods are detailed in the Supplementary Appendix, including plausibility screening, handling of artefact and missingness, and minimum-data criteria for cumulative exposure measures. Exposures were prespecified and retained in all models. Downstream mediators, for example treatments triggered by post-ROSC physiology, were excluded. Continuous variables are summarised as mean (standard deviation) or median (interquartile range), and categorical variables as count (percent).

Minute level exposures, SBP, MAP, SpO_2_, ETCO_2_, RR, were modelled as continuous variables. Anticipated nonlinearity was addressed with fractional polynomials using the Royston and Sauerbrei multivariable approach.^15^ Models were first univariate to assess functional form, then extended to multivariable logistic regression. Fractional polynomial terms were retained when likelihood ratio tests improved fit.

A directed acyclic graph identified age, sex, initial rhythm, downtime, witnessed status, arrest location, comorbidity, cause of arrest, and resuscitation drugs as confounders of the relation between post-ROSC physiology and outcomes (Fig. S1). Measured confounders were included in all multivariable models; comorbidity, which was unavailable, was addressed using sensitivity analyses.^16^ These covariates entered all multivariable models regardless of statistical significance. We used multivariable logistic regression with robust standard errors. We report adjusted odds ratios with 95% confidence intervals using complete-case analyses.

Because comorbidity was identified as a confounder in the causal model but was unavailable for adjustment, we performed sensitivity analyses to assess its potential impact. Comorbidity adjustment attenuates effects 9.5% toward the null.^17^ Sensitivity analyses used Greenland’s framework.^18^ Analyses derived the confounder imbalance to nullify effects, applied 9.5 percent log-odds shrinkage, and computed E values.^19^

### Ethics

Ethics approval was obtained from the Monash University Human Research Ethics Committee (ID 41476). Ambulance Victoria Research Committee approved the study (R24-001).

## Results

### Cohort

**Fig. 1:**
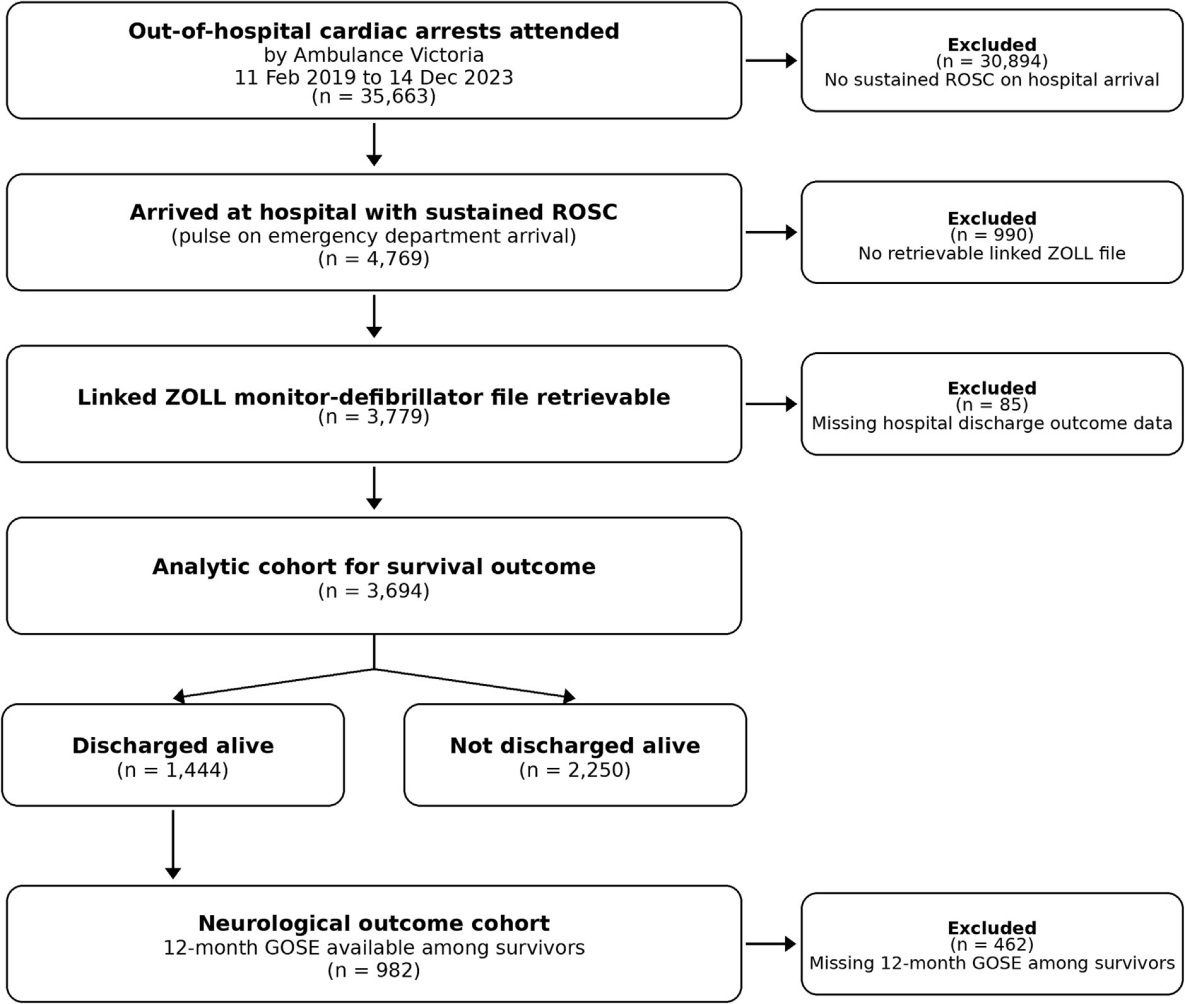
**Study cohort**. Adults with out-of-hospital cardiac arrest attended by Ambulance Victoria between 11 February 2019 and 14 December 2023 (*n* = 4769 with sustained ROSC at ED handover).

Ambulance Victoria attended 35,663 OHCAs (Fig. 1). Of these, 4769 arrived at hospital with a pulse, and 3779 had a retrievable Zoll® file. After excluding 85 without outcomes, the analytic cohort was 3694. The median time from recorded ROSC to hospital arrival was 58 min (IQR 44–75). Twelve-month GOSE was available for 982 survivors. Missingness was <1% for Utstein variables. Within linked monitor recordings (*n* = 3779), SBP, MAP and SpO_2_ streams were available for 99.6%, 99.5% and 98.9% of patients, while capnography-derived ETCO_2_ and RR streams were unavailable in 11.9% and 11.9%. Some derived measures, particularly mean values requiring sufficient minute-level data, had additional missingness, resulting in exposure-specific model sample sizes (Table S4).

### Baseline characteristics

Overall, 1444 (39.1%) patients survived (Table 1). OHCAs occurred at home in 66.0% of cases and were bystander witnessed in 56.0%. Excluding EMS witnessed events, bystander CPR occurred in 80.0%. A shockable rhythm was present in half of cases, and a presumed cardiac cause in 76.5%. The primary medications given were adrenaline (80.2%) amiodarone (20.1%). Airway management was often sequential, with ETI and SGA used in the same patient in 65.8%. ETI was used in 76.5% and SGA in 75.3%, and 14.0% were managed with bag-valve-mask alone, with no advanced airway. Bag-valve-mask only management was much more common among survivors than non-survivors (31.4% vs 2.8%).

**Table 1:** Baseline characteristics of OHCA patients with sustained ROSC, Victoria 2019–2023.

**Variable**	**Total (*N* = 3694)**	**Survivors (*N* = 1444)**	**Non-survivors (*N* = 2250)**
Age (years)	3694; 63 (51–75)	1444; 59 (49–70)	2250; 67 (53–78)
Downtime (min)	3688; 28 (21–40)	1439; 22 (16–35)	2249; 32 (24–41)

**Sex**			
Male	2558 (69.3)	1075 (74.5)	1483 (66.0)
Female	1133 (30.7)	369 (25.6)	764 (34.0)

**Location of arrest**			
Home/residence	2439 (66.0)	774 (53.6)	1665 (74.0)
Public place	834 (22.6)	455 (31.5)	379 (16.8)
Other	421 (11.4)	215 (14.9)	206 (9.2)

**Witnessed status**			
EMS witnessed	860 (23.3)	484 (33.5)	376 (16.8)
Not witnessed	762 (20.7)	165 (11.4)	597 (26.6)
Public witness	2064 (56.0)	795 (55.1)	1269 (56.6)

**Bystander CPR**			
Yes	2260 (80.0)	833 (86.8)	1427 (76.5)
No	566 (20.3)	127 (13.2)	439 (23.5)

**Initial rhythm**			
VF/VT	1858 (50.4)	1162 (80.8)	696 (31.0)
PEA	956 (25.9)	221 (15.4)	735 (32.7)
Asystole	749 (20.3)	42 (2.9)	707 (31.5)
Other/unknown	122 (3.3)	14 (1.0)	108 (4.8)

**Presumed cause**			
Cardiac	2824 (76.5)	1268 (87.8)	1556 (69.1)
Respiratory	317 (8.6)	56 (3.9)	261 (11.6)
Overdose/poisoning	166 (4.5)	52 (3.6)	114 (5.1)
Trauma	125 (3.4)	25 (1.7)	100 (4.4)
Asphyxial	108 (2.9)	12 (0.8)	96 (4.3)
Medical	129 (3.5)	22 (1.5)	107 (4.8)
Other	25 (0.6)	9 (0.6)	16 (0.7)

**Drugs administered**			
Adrenaline	2964 (80.2)	818 (56.7)	2146 (95.4)
Amiodarone	744 (20.1)	347 (24.0)	397 (17.6)

**Airway intervention**			
ETI	2826 (76.5)	769 (53.3)	2057 (91.4)
Supraglottic airway	2783 (75.3)	848 (58.7)	1935 (86.0)
Bag-valve-mask only	516 (14.0)	454 (31.4)	62 (2.8)

Values are median (IQR) or *n* (%). Percentages in the Survivors and Non-survivors columns are column percentages. Survivors were discharged alive. Sustained ROSC denotes palpable pulses at emergency department handover. Denominators reflect available data per column. Bystander CPR excludes EMS-witnessed arrests, consistent with Utstein definitions.

### Post-ROSC physiological parameters

**Fig. 2:**
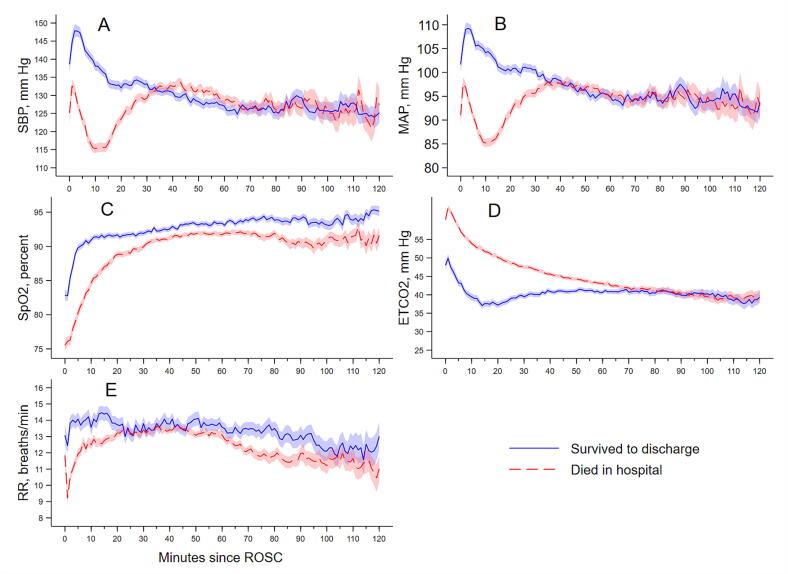
**Minute-by-minute mean physiological values after ROSC**. (A) Systolic blood pressure (SBP), (B) Mean arterial pressure (MAP), (C) Peripheral oxygen saturation (SpO_2_), (D) End-tidal carbon dioxide (ETCO_2_), and (E) Respiratory rate (RR). Shaded regions represent 95% confidence intervals. Curves reflect available monitor data only, minutes after monitoring ends are treated as missing and values are not carried forward. ETCO_2_ and RR are capnography-derived and may be affected by airway type and seal, ventilation strategy, and pulmonary perfusion, so they should not be interpreted as treatment targets.

After de-duplication, the median number of distinct readings per patient were: HR = 201, SpO_2_ = 97, blood pressure = 36, ETCO_2_ = 150, and RR = 103. Table S1 and Fig. 2 summarise post-ROSC physiology. Systolic and mean arterial pressures were higher in survivors than in non-survivors, with blood pressure rising during the first 5–10 min after ROSC before reaching a plateau. Episodes of hypotension were common, particularly among non-survivors. Oxygen saturation increased rapidly after ROSC, though transient hypoxaemia was frequent. End-tidal CO_2_ values were lower in survivors and decreased gradually during the monitoring period. Respiratory rate changed relatively little over time. Together, these measurements depict the early post-ROSC period as one of circulatory and oxygenation recovery in the prehospital phase.

### Post-ROSC physiological parameters and survival

Of 3779 with retrievable monitor files, 3694 had complete survival outcome data and were included in analyses; neurological analyses were restricted to survivors with 12-month GOSE available. Sample size varied across physiology models due to missing physiologic streams (Tables 2, S2; Table S4). Predicted probabilities are in Figs. S2–S11.

**Table 2:** Adjusted associations between post-ROSC physiological parameters and survival to discharge.

**Physiological parameters and model type**	**Adjusted OR (95% CI)**	***p*-value**
**Systolic blood pressure (mm Hg)**		
*Mean SBP (mm Hg), ref = 100*		0.002
80 vs 100	0.87 (0.79–0.95)	
120 vs 100	1.15 (1.05–1.26)	
140 vs 100	1.32 (1.11–1.59)	
*Minimum SBP (mm Hg), ref = 100*		<0.001
80 vs 100	0.72 (0.65–0.80)	
120 vs 100	1.35 (1.21–1.50)	
140 vs 100	1.77 (1.44–2.18)	
*Minutes with SBP <90, ref = 0 min*		<0.001
10 min vs 0 min	0.58 (0.49–0.70)	
30 min vs 0 min	0.44 (0.35–0.57)	
60 min vs 0 min	0.38 (0.29–0.51)	

**Mean arterial pressure (mm Hg)**		
*Mean MAP (mm Hg), ref = 75*		0.003
65 vs 75	0.91 (0.85–0.97)	
90 vs 75	1.15 (1.05–1.27)	
100 vs 75	1.27 (1.09–1.48)	
*Minimum MAP (mm Hg), ref = 75*		<0.001
65 vs 75	0.79 (0.74–0.85)	
90 vs 75	1.34 (1.18–1.53)	
100 vs 75	1.50 (1.16–1.95)	
*Minutes with MAP <65, ref = 0 min*		<0.001
10 min vs 0 min	0.56 (0.47–0.67)	
30 min vs 0 min	0.42 (0.33–0.54)	
60 min vs 0 min	0.37 (0.29–0.49)	

**Oxygen saturation (SpO_2_, %)**		
*Mean SpO_2_ (%), ref = 95*		<0.001
80 vs 95	0.45 (0.35–0.58)	
90 vs 95	0.70 (0.63–0.79)	
98 vs 95	1.07 (1.04–1.09)	
*Minimum SpO_2_ (%), ref = 95*		<0.001
80 vs 95	0.65 (0.55–0.76)	
90 vs 95	0.76 (0.68–0.84)	
98 vs 95	1.13 (1.08–1.18)	
*Minutes with SpO_2_ <90%, ref = 0 min*		<0.001
10 min vs 0	0.95 (0.93–0.98)	
30 min vs 0	0.87 (0.81–0.94)	
60 min vs 0	0.77 (0.67–0.89)	

**End-tidal CO_2_ (mm Hg)**		
*Mean ETCO_2_ (mm Hg), ref = 40*		<0.001
30 vs 40	1.56 (1.40–1.74)	
50 vs 40	0.63 (0.57–0.70)	
60 vs 40	0.41 (0.34–0.50)	
*Minimum ETCO_2_ (mm Hg), ref = 20*		<0.001
10 vs 20	1.18 (1.11–1.25)	
30 vs 20	0.76 (0.69–0.83)	
40 vs 20	0.54 (0.44–0.66)	
*Minutes with ETCO_2_ <20, ref = 0 min*		<0.001
5 vs 0	1.37 (1.13–1.65)	
10 vs 0	1.50 (1.19–1.88)	
20 vs 0	1.64 (1.28–2.10)	

**Respiratory rate (breaths/min)**		
*Mean RR (breaths/min), ref = 12*		<0.001
8 vs 12	0.44 (0.36–0.54)	
10 vs 12	0.68 (0.62–0.75)	
18 vs 12	2.35 (1.95–2.83)	
*Minimum RR (breaths/min), ref = 12*		<0.001
6 vs 12	0.38 (0.30–0.48)	
10 vs 12	0.73 (0.67–0.79)	
20 vs 12	2.99 (2.18–4.11)	
*Minutes with RR <8, ref = 0 min*		<0.001
5 vs 0 min	0.35 (0.28–0.43)	
10 vs 0 min	0.32 (0.25–0.40)	
20 vs 0 min	0.30 (0.23–0.39)	
*Minutes with RR >30, ref = 0 min*		0.01
10 vs 0 min	1.30 (1.05–1.60)	
20 vs 0 min	1.68 (1.11–2.55)	
30 vs 0 min	2.18 (1.17–4.07)	

Adjusted for age, sex, downtime, initial rhythm, witnessed status, arrest location, presumed cause, and resuscitation drugs (adrenaline, amiodarone). Robust standard errors were applied at the patient level. Continuous variables were modelled using prespecified fractional-polynomial functional forms derived from univariate analyses (see Supplementary Table S3). *P*-values represent joint Wald tests for the continuous terms, and adjusted odds ratios were estimated post-estimation using margins contrasts at selected values relative to the reference. Analytic cohort comprised 3694 patients with complete outcome data. Model sample size varied by exposure due to missing physiologic streams (see Table S4). ETCO_2_ and RR are capnography-derived measures and are influenced by airway seal and ventilation strategy, so associations should be interpreted as context-dependent markers rather than treatment targets.

#### Systolic blood pressure

Higher post-ROSC systolic pressure was independently associated with improved survival (Table 2). Relative to 100 mm Hg, adjusted odds rose from 0.87 (0.79–0.95) at 80 mm Hg to 1.32 (1.11–1.59) at 140 mm Hg. Minimum systolic pressure showed the same pattern, and longer time with SBP <90 mm Hg corresponded to lower survival. Fig. S2 shows increasing probability of survival with higher systolic pressure and declining survival probability with longer hypotension. Systolic pressure showed a modest positive association with good neurological outcome (Fig. S3).

#### Mean arterial pressure

Higher mean and minimum MAP were consistently associated with greater odds of survival (Table 2). Relative to 75 mm Hg, survival odds were lower at 65 mm Hg and higher at 90–100 mm Hg. Longer time with MAP <65 mm Hg corresponded to steady declines in the probability of survival. Fig. S4 shows near linear gains in survival probability with rising MAP and wider uncertainty at extremes. MAP measures showed weak and inconsistent associations with 12-month neurological outcome, with no clear monotonic pattern across the range (Fig. S5; Table S2).

#### End-tidal CO_2_

ETCO_2_ values were lower in survivors than non-survivors (Table S1; Fig. 2D). In adjusted models, predicted survival declined as mean and minimum ETCO_2_ increased across the observed range (Table 2; Fig. S6A, B). However, ETCO_2_ depends on airway seal and ventilation strategy, and airway management was often sequential. In this cohort, bag-valve-mask only management was much more common among survivors, which can bias ETCO_2_ low through leak. For this reason, the direction of the ETCO_2_ threshold-time analysis (minutes with ETCO_2_ <20 mm Hg) should not be interpreted as a protective effect of low CO_2_ and is presented as a descriptive association only (Fig. S6C).

#### Oxygen saturation

Survival decreased as SpO_2_ fell, highest near 95–100% and much lower at 80–85% (Table 2). Longer time below 90% further separated outcomes, with prolonged desaturation linked to lower survival. Fig. S8 shows these graded relations. No oxygenation measure was associated with 12-month neurological recovery, with flat curves across the range (Fig. S9).

#### Respiratory rate

RR was derived from the capnography channel. In ventilated patients it largely reflects delivered ventilation, while in others it may reflect spontaneous breathing. In adjusted models, RR showed a modest nonlinear association with survival (Table 2; Fig. S10), with lower survival at very low rates. Higher RR and time with RR > 30 were associated with higher survival, which is most consistent with spontaneous breathing or lighter sedation in some patients rather than a benefit of targeting tachypnoea. None of the respiratory-rate measures were significantly associated with 12-month neurological outcomes (Fig. S11).

### Sensitivity analysis

We applied a 9.5% toward-null attenuation to each estimated ratio measure (odds ratio) from the mean post-ROSC physiology models, as a sensitivity analysis for potential unmeasured confounding by comorbidity. No effect reversed or approached the null, and the overall pattern remained stable. E values ranged from 3 to 5, indicating a confounder would need strong joint associations with physiology and survival to explain the findings.

## Discussion

This analysis shows that survival after ROSC increases with higher systolic and mean arterial pressures and with greater oxygen saturation. Across parameters, survival tracked with return toward homeostatic values. Neurological outcomes showed a small association with mean SBP, while MAP, SpO_2_, ETCO_2_, and respiratory rate showed weak relationships without a clear dose–response pattern. This dissociation suggests that early physiological stability may be necessary for survival, yet not sufficient for meaningful neurological recovery. Brain injury remains the leading cause of death after admission. Consistent with this, guidelines prioritise temperature normalisation, avoidance of hypoxaemia and hypotension, maintenance of normocapnia, and structured neuroprognostication.^3^, ^14^, ^20^, ^21^ Together, these elements highlight that early circulatory stability, normalisation of ventilation, and avoidance of hypoxaemia keep patients alive long enough for in-hospital determinants of neurological recovery, such as hypoxic-ischaemic brain injury severity, seizure control, temperature management, and ICU haemodynamic care, to influence outcome.

A critical point is that our neurological analysis includes only survivors. This likely explains weaker associations with neurological outcomes. To expand on this context, early haemodynamic stability after ROSC was associated with better survival. This period is characterised by vasoplegia, myocardial dysfunction, and impaired autoregulation, such that even small increments in blood pressure can improve coronary and cerebral perfusion.^20^ Consistent with this pattern, registry data indicate that circulatory shock at hospital admission independently predicts poorer neurological recovery.^21^ Evidence from animal and human post-arrest studies shows loss of autoregulatory buffering, with cerebral blood flow becoming pressure dependent.^22^ In line with these mechanisms, our data show that brief hypotension was associated with worse survival, and loss of perfusion may trigger irreversible injury. The ICU BOX trial showed no difference in death or neurological outcome at 90 days with MAP targets.^8^ This absence of effect may relate to the timing of intervention, which began in the ICU rather than in the first post-ROSC minutes.

Measurement of blood pressure within minutes of ROSC accounts for the higher prevalence of hypotension observed in this cohort. Unlike studies that rely on later readings, many of our measurements occur before fluids or vasopressors have had time to restore vascular tone. Our data therefore capture the early peak of shock, when myocardial stunning, vasoplegia, and hypovolaemia contribute to circulatory collapse.^20^ This timing matters for oxygen delivery, because saturation and perfusion both determine oxygen delivery, and early pressure instability can blunt the benefit of good oxygenation.

Building on prior work, earlier investigations of perfusion pressure after ROSC were limited to sparse or single-point measurements, which restricted insight into how blood pressure changes during the earliest post-ROSC minutes. Prior blood pressure studies identified post-ROSC hypotension as a predictor of mortality, but most used single handover readings rather than continuous monitoring.^23^, ^24^ Randomised trials have not shown survival or neurological benefit from higher MAP targets.^8^ Taken together, this leaves uncertainty about whether risk is driven by a threshold effect or by cumulative exposure across the blood pressure range. By contrast, our analysis captures the transition period immediately after ROSC and shows that even transient hypotension during the first post-ROSC minutes is associated with lower survival.

Studies that used the final prehospital reading at handover often reported a U‑shaped association.^23^ That pattern is likely influenced by timing and treatment rather than reflecting a true physiologic optimum. A single snapshot can mix patients who were initially hypotensive but improving with others whose pressures were increased by fluids or vasopressors. Our analysis used continuous and time-averaged values within the early post-ROSC window to capture each patient’s haemodynamic exposure. This approach yielded a largely monotonic relation in which worsening physiology tracked with lower survival. This methodological distinction helps reconcile differences across studies and supports time‑based aggregate measures over isolated readings.

Oxygenation findings complement these trends. Early oxygen delivery helps restore normal oxygen delivery after ischaemia.^20^ Titration timing is crucial, as early prehospital down-titration can be too soon, while ICU-only titration may be too late.^25^ Our data capture the early post-ROSC period, with SpO_2_ rising and stabilising. Prehospital management commonly begins with high inspired oxygen immediately after ROSC, with titration once monitoring is stable to avoid hypoxaemia. In this setting, low SpO_2_ is best interpreted as oxygenation failure and illness severity despite treatment rather than lower oxygen dose, and high SpO_2_ cannot distinguish degrees of hyperoxaemia.^3^ This aligns with trials comparing higher and conservative oxygen targets show no clear benefit in the ICU, while restrictive prehospital targets were associated with lower survival.^8^, ^10^, ^25^

Capnography measures need careful interpretation in this setting. In our adjusted models, higher ETCO_2_ values were associated with lower survival, but ETCO_2_ and capnography-derived RR are shaped by ventilation strategy, airway type and seal, pulmonary perfusion, and illness severity. Very low ETCO_2_ values (for example around 10 mm Hg) can occur early after ROSC with low pulmonary blood flow or airway leak, and do not necessarily indicate extreme hypocapnia. In this cohort, airway management differed strongly by outcome, with bag-valve-mask only management far more common among survivors, which can bias ETCO_2_ low. Time-below-threshold measures are also sensitive to monitoring duration and censoring. Taken together, the observed ETCO_2_ and RR associations are best interpreted as context-dependent markers of early post-ROSC state rather than effects of changing ventilation targets.

Finally, emerging evidence indicates that dynamic trends, rather than isolated values, predict outcome, and that concurrent elevations in SBP, SpO_2_, and ETCO_2_ during the immediate post-resuscitation phase are linked with higher survival.^23^ Taken together, these studies emphasise that recovery depends on the coordination of multiple systems, not on meeting cutoffs. Overall, generalisability is restricted to similar EMS systems, and residual confounding cannot be excluded. These findings argue for a more trajectory-based approach to post-resuscitation care, focuses on maintaining coordination rather than chasing isolated numerical goals.

### Limitations

The primary strength is the use of minute-by-minute physiological recordings, which let us capture early post-ROSC changes and model non-linear associations. Although we simplified this detailed data into per-patient summary numbers, this was intentional to provide clinically interpretable summaries. Observational design limits causal inference, and treatments (fluids, vasopressors, ventilation settings, sedation) were clinician-directed and not standardised. Monitoring can end at transport completion or handover, so later minutes are missing rather than carried forward. ETCO_2_ and capnography-derived RR depend on airway seal, ventilation strategy, and pulmonary blood flow, and we could not classify airway type, ventilation mode, or sedation minute-by-minute in a cohort where airway management was often sequential. These findings should therefore be interpreted as context-dependent associations rather than physiologic targets. Meaningful residual confounding by comorbidity appears unlikely, but unmeasured confounding may remain.

## Conclusion

Survival after out-of-hospital cardiac arrest was higher in patients with higher early post-ROSC blood pressure and oxygen saturation, and lower with longer exposure to hypotension or hypoxaemia. Capnography-derived ETCO_2_ and RR showed associations with outcome that likely reflect airway and ventilation context and illness severity and should be interpreted as context markers rather than treatment targets. These findings support using early minute-level trajectories to inform monitoring and trial design in the immediate post-ROSC period.

## Data sharing statement

This study is a registry-based analysis of linked Ambulance Victoria and Zoll® monitor data. All aggregated data relevant to the study are included in the article or uploaded as supplementary information. De-identified raw data are not publicly available due to privacy and governance restrictions but may be available from the corresponding author upon reasonable request and subject to approval by Ambulance Victoria and relevant ethics committees.

## CRediT authorship contribution statement

**Pieter Francsois Fouche:** Writing – review & editing, Writing – original draft, Methodology, Formal analysis, Conceptualization. **Emily Nehme:** Writing – review & editing, Project administration, Methodology, Formal analysis, Data curation. **Sam Burton:** Writing – review & editing, Data curation. **Belinda Flanagan:** Writing – review & editing. **Benjamin Meadley:** Writing – review & editing, Investigation. **David Anderson:** Writing – review & editing, Resources, Conceptualization. **Dion Stub:** Writing – review & editing. **Ziad Nehme:** Writing – review & editing, Writing – original draft, Supervision, Resources, Project administration, Investigation, Conceptualization.

## Funding

This research did not receive any specific grant from funding agencies in the public, commercial, or not-for-profit sectors.

## Declaration of competing interest

The authors declare that they have no known competing financial interests or personal relationships that could have appeared to influence the work reported in this paper.
