# Supplementary material for: High-fidelity minute-level physiologic trajectories after ROSC from linked monitor-defibrillator recordings in out-of-hospital cardiac arrest
Source: Resusc Plus. 2026 Mar 6;28:101286. doi: 10.1016/j.resplu.2026.101286 (PMC12991840; doi:10.1016/j.resplu.2026.101286)
Supplement: Supplementary Data 1 [file mmc1.docx]

*Table S1 Post-ROSC physiological exposures during prehospital monitoring*

| **Variable** | | | **Total (N = 3,694)** | **Survivors (N = 1,444)** | **Non-survivors (N = 2,250)** |
| --- | --- | --- | --- | --- | --- |
| **Systolic Blood Pressure (mm Hg)** | | |  |  |  |
| SBP (median of patient means) | | | 124 (110–137) | 127 (115–141) | 121 (106–135) |
| SBP minimum | | | 72 (57–91) | 86 (67–106) | 65 (52–82) |
| Any SBP <90 mm Hg | | | 2696 (73.0) | 807 (55.9) | 1889 (84.0) |
| Minutes SBP <90 mm Hg | | | 15 (0–37) | 5 (0–20) | 23 (8–47) |
| **Mean Arterial Pressure (mm Hg)** | | |  |  |  |
| MAP (median of patient means) | | | 92 (82–102) | 95 (86–105) | 90 (79–100) |
| MAP minimum | | | 54 (42–69) | 65 (50–79) | 50 (40–61) |
| Any MAP <65 mm Hg | | | 2513 (68.0) | 714 (49.4) | 1799 (80.0) |
| Minutes MAP <65 mm Hg | | | 11 (0–30) | 0 (0–14) | 18 (5–39) |
| **Oxygen saturation (%)** | | |  |  |  |
| SPO₂ mean | | | 91 (85–94) | 93 (89–96) | 89 (83–93) |
| SPO₂ minimum | | | 71 (59–80) | 77 (66–82) | 66 (56–77) |
| Any SpO₂ <90 % | | | 3411 (92.3) | 1293 (89.5) | 2118 (94.1) |
| Minutes SpO₂ <90 % | | | 66 (22–128) | 40 (12–87) | 86 (34–146) |
| **End-tidal CO₂ _(_mm Hg)** | | |  |  |  |
| ETCO₂ mean | | | 43 (35–52) | 37 (31–43) | 46 (38–56) |
| ETCO₂ minimum | | | 25 (14–34) | 20 (11–29) | 28 (17–36) |
| Any ETCO₂ <20 mm Hg | | | 1189 (36.7) | 514 (49.4) | 675 (30.7) |
| Minutes ETCO₂ <20 mm Hg | | | 0 (0–7) | 0 (0–14) | 0 (0–4) |
| **Respiratory rate (breaths/min)** | | |  |  |  |
| RR mean | | | 14 (11–17) | 16 (14–19) | 13 (11–15) |
| RR minimum | | | 7 (5–8) | 8 (6–10) | 6 (5–8) |
| RR maximum | | | 24 (19–30) | 27 (22–32) | 23 (18–29) |
| Any RR <8 or >30 breaths/min | | | 2825 (76.5) | 1077 (74.6) | 1748 (77.7) |
| Minutes RR outside 8–30 | | | 1 (0–5) | 0 (0–3) | 2 (0–7) |
|  |  | |  |  |  |

Values are median (IQR) or number (percent). Exposures summarise all available minute-level monitoring after ROSC. SBP and MAP represent the median of patient-level mean values. Thresholds: SBP < 90 mm Hg, MAP < 65 mm Hg, SpO₂ < 90%, ETCO₂ < 20 mm Hg, RR < 8 or > 30 breaths/min. Denominators reflect available data per column.

*Table S2 Adjusted associations between post-ROSC physiological parameters and 12-month good neurological outcome among survivors to hospital discharge*

| **Physiological parameter and model type** | | | | **Adjusted OR (95 % CI)** | **p-value** | |
| --- | --- | --- | --- | --- | --- | --- |
| **Systolic Blood Pressure (mm Hg)** | | | |  |  | |
| *Mean SBP (mm Hg), ref = 100* | | | |  | 0.02 | |
| 80 vs 100 | | | | 0.75 (0.61 – 0.92) |  | |
| 120 vs 100 | | | | 1.26 (1.05–1.52) |  | |
| 140 vs 100 | | | | 1.40 (1.02–1.93) |  | |
| *Minimum SBP (mm Hg), ref = 100* | | | |  | 0.18 | |
| 80 vs 100 | | 0.99 (0.87–1.13) | |  |  |  |
| 120 vs 100 | | 0.92 (0.80–1.07) | |  |  |  |
| 140 vs 100 | | 0.74 (0.51–1.06) | |  |  |  |
| *Minutes with SBP < 90, ref = 0 min* | | | |  | 0.007 | |
| 10 min vs 0 min | | 0.99 (0.99–1.00) | |  |  |  |
| 30 min vs 0 min | | 0.99 (0.98 – 1.00) | |  |  |  |
| 60 min vs 0 min | | 0.92 (0.86–0.98) | |  |  |  |
| **Mean Arterial Pressure (mm Hg)** | | | |  |  | |
| *Mean MAP (mm Hg), ref = 75* | | | |  | 0.17 | |
| 65 vs 75 | | | | 0.80 (0.68–0.94) |  | |
| 90 vs 75 | | | | 1.18 (0.98–1.42) |  | |
| 100 vs 75 | | | | 1.25 (0.94–1.64) |  | |
| *Minimum MAP (mm Hg), ref = 75* | | | |  | 0.32 | |
| 65 vs 75 | | | | 1.04 (0.96 – 1.12) |  | |
| 90 vs 75 | | | | 0.94 (0.84–1.06) |  | |
| 100 vs 75 | | | | 0.91 (0.75–1.10) |  | |
| *Minutes with MAP < 65, ref = 0 min* | | | |  | 0.29 | |
| 10 min vs 0 min | | | | 0.98 (0.94–1.02) |  | |
| 30 min vs 0 min | | | | 0.94 (0.84–1.05) |  | |
| 60 min vs 0 min | | | | 0.89 (0.71–1.11) |  | |
| **Oxygen saturation (SpO₂, %)** | | | |  |  | |
| *Mean SpO₂ (%), ref = 95* | | | |  | 0.87 | |
| 85 vs 95 | | | | 0.98 (0.75 – 1.28) |  | |
| 90 vs 95 | | | | 0.99 (0.87 – 1.13) |  | |
| 98 vs 95 | | | | 1.01 (0.93 – 1.09) |  | |
| *Minimum SpO₂ (%), ref = 95* | | | |  | 0.90 | |
| 80 vs 95 | | | | 0.99 (0.81 – 1.20) |  | |
| 90 vs 95 | | | | 1.00 (0.93 – 1.06) |  | |
| 98 vs 95 | | | | 1.00 (0.94 – 1.07) |  | |
| *Minutes with SpO₂ < 90 %, ref = 0 min* | | | |  | 0.70 | |
| 10 min vs 0 | | | | 1.00 (0.97 – 1.02) |  | |
| 30 min vs 0 | | | | 0.99 (0.92–1.06) |  | |
| 40 min vs 0 | | | | 0.98 (0.90–1.08) |  | |
| **End-tidal CO₂ _(_mm Hg)** | | | |  |  | |
| *Mean ETCO₂ (mm Hg), ref = 40* | | | |  | 0.78 | |
| 30 vs 40 | | | | 1.02 (0.88–1.19) |  | |
| 50 vs 40 | | | | 0.98 (0.84–1.12) |  | |
| 60 vs 40 | | | | 0.96 (0.71–1.29) |  | |
| *Minimum ETCO₂ (mm Hg), ref = 20* | | | |  | 0.69 | |
| 10 vs 20 | | | | 1.03 (0.88–1.21) |  | |
| 30 vs 20 | | | | 0.97 (0.84–1.13) |  | |
| 40 vs 20 | | | | 0.94 (0.70–1.27) |  | |
| *Minutes with ETCO₂ < 20, ref = 0 min* | | | |  | 0.12 | |
| 5 vs 0 | | | | 1.00 (1.00–1.00) |  | |
| 10 vs 0 | | | | 1.00 (1.00–1.00) |  | |
| 20 vs 0 | | | | 0.99 (0.97–1.00) |  | |
| **Respiratory rate (breaths/min)** | | | |  |  | |
| *Mean RR (breaths/min), ref = 12* | | | |  | 0.74 | |
| 8 vs 12 | | | | 0.98 (0.85 – 1.12) |  | |
| 10 vs 12 | | | | 0.99 (0.92 – 1.06) |  | |
| 18 vs 12 | | | | 1.04 (0.79 – 1.39) |  | |
| *Minimum RR (breaths/min), ref = 12* | | | |  | 0.53 | |
| 6 vs 12 | | | | 0.93 (0.73 – 1.18) |  | |
| 10 vs 12 | | | | 0.97 (0.90 – 1.06) |  | |
| 20 vs 12 | | | | 1.11 (0.80 – 1.52) |  | |
| *Minutes with RR < 8, ref = 0 min* | | | |  | 0.35 | |
| 5 vs 0 min | | | | 1.07 (0.93 – 1.23) |  | |
| 10 vs 0 min | | | | 1.14 (0.87 – 1.50) |  | |
| 20 vs 0 min | | | | 1.30 (0.75 – 2.25) |  | |
| *Minutes with RR > 30, ref = 0 min* | | | |  | 0.84 | |
| 10 vs 0 min | | | | 0.97 (0.74 – 1.28) |  | |
| 20 vs 0 min | | | | 0.95 (0.55 – 1.63) |  | |
| 30 vs 0 min | | | | 0.92 (0.41 – 2.09) |  | |
|  | |  | |  | |  |

Adjusted for age, sex, downtime, initial rhythm, witnessed status, arrest location, presumed cause, and resuscitation drugs (adrenaline, amiodarone). Analyses were limited to patients discharged alive with available 12-month neurological outcomes (N = 982). Continuous variables were modelled using prespecified fractional-polynomial functional forms. P-values represent joint Wald tests for continuous terms, and adjusted odds ratios were estimated post-estimation using margins contrasts.

*Supplementary Table S3. Functional forms used in multivariable survival models*

| Variable | Outcome | Final FP Functional Form | Expression (generated form) |
| --- | --- | --- | --- |
| Age (years) | Adjustment | FP2(2,3) | X², X³ |
| Downtime (min) | Adjustment | FP2(–0.5, –0.5) | X^–0.5, X^–0.5·ln(X) |
| SBP (mean) | Survival | Linear | X |
| SBP (minimum) | Survival | FP2(−0.5, 0) | X-0.5, X·ln(X) |
| Minutes SBP <90 | Survival | FP2(0.5,0.5) | X^0.5, X^0.5·ln(X) |
| MAP (mean) | Survival | Linear | X |
| MAP (minimum) | Survival | FP2(3,3) | X³, X³·ln(X) |
| Minutes MAP <65 | Survival | FP2(0.5,0.5) | X^0.5, X^0.5·ln(X) |
| ETCO₂ (mean) | Survival | FP2(2,3) | X², X³ |
| ETCO₂ (minimum) | Survival | FP2(0, 0.5) | ln(X) and X^0.5 |
| Minutes ETCO₂ <20 | Survival | FP2(0.5, 0.5) | X^0.5, X^0.5·ln(X) |
| SpO₂ (mean) | Survival | FP1(3) | X³ |
| SpO₂ (minimum) | Survival | FP1(2) | X² |
| Minutes SpO₂ <90 | Survival | FP2(1,2) | X, X² |
| RR (mean) | Survival | FP2(1,2) | X, X² |
| RR (minimum) | Survival | FP2(–0.5,0) | X^–0.5, ln(X) |
| Minutes RR <8 | Survival | FP1(–1) | X^–1 |
| Minutes RR >30 | Survival | Linear | X |
| SBP (mean) | Neuro | FP2(3,3) | X³, X³·ln(X) |
| SBP (minimum) | Neuro | FP2(3,3) | X³, X³·ln(X) |
| Minutes SBP <90 | Neuro | FP2(3,3) | FP2(3,3) |
| MAP (mean) | Neuro | FP2(2,3) | X², X³ |
| MAP (minimum) | Neuro | Linear | X |
| Minutes MAP <65 | Neuro | Linear | X |
| ETCO₂ (mean) | Neuro | Linear | X |
| ETCO₂ (minimum) | Neuro | Linear | X |
| Minutes ETCO₂ <20 | Neuro | FP1(3). | X³ |
| SpO₂ (mean) | Neuro | Linear | X |
| SpO₂ (minimum) | Neuro | Linear | X |
| Minutes SpO₂ <90 | Neuro | Linear | X |
| RR (mean) | Neuro | Linear | X |
| RR (minimum) | Neuro | Linear | X |
| Minutes RR <8 | Neuro | Linear | X |
| Minutes RR >30 | Neuro | Linear | X |

Fractional-polynomial transformations were selected using the Royston–Sauerbrei multivariable fractional-polynomial (MFP) procedure based on model fit criteria. The final functional forms for each exposure were carried forward into multivariable models for survival and neurological outcomes. Complete-case analysis (N = 3,694)

*Table S4 Model diagnostics for multivariable logistic regression models*

| Outcome | Exposure | Model specification | N | AUC (ROC) | Correctly classified (%) | Hosmer–Lemeshow p-value | Pseudo R^2^ |
| --- | --- | --- | --- | --- | --- | --- | --- |
| Survival | SBP | Mean | 3,444 | 0.90 | 82.6 | 0.74 | 0.43 |
| Survival | SBP | Minimum | 3,444 | 0.91 | 83.4 | 0.94 | 0.44 |
| Survival | SBP | Min. < 90 mm Hg | 3,460 | 0.91 | 83.2 | 0.36 | 0.55 |
| Neuro | SBP | Mean | 913 | 0.65 | 67.5 | 0.04 | 0.06 |
| Neuro | SBP | Minimum | 913 | 0.65 | 67.4 | 0.44 | 0.06 |
| Neuro | SBP | Min. < 90 mm Hg | 917 | 0.64 | 67.7 | 0.17 | 0.06 |
| Survival | MAP | Mean | 3,442 | 0.90 | 82.5 | 0.88 | 0.43 |
| Survival | MAP | Minimum | 3,442 | 0.91 | 83.2 | 0.74 | 0.44 |
| Survival | MAP | Min. < 65 mm Hg | 3,460 | 0.91 | 82.9 | 0.03 | 0.44 |
| Neuro | MAP | Mean | 913 | 0.65 | 67.8 | 0.65 | 0.05 |
| Neuro | MAP | Minimum | 913 | 0.64 | 67.1 | 0.45 | 0.05 |
| Neuro | MAP | Min. < 65 mm Hg | 917 | 0.64 | 67.5 | 0.33 | 0.05 |
| Survival | ETCO₂ | Mean | 3,017 | 0.89 | 82.9 | 0.42 | 0.39 |
| Survival | ETCO₂ | Minimum | 3,017 | 0.88 | 82.2 | 0.77 | 0.37 |
| Survival | ETCO₂ | ETCO₂<20 mm Hg | 3,017 | 0.88 | 82.2 | 0.76 | 0.37 |
| Neuro | ETCO₂ | Mean | 642 | 0.66 | 67.0 | 0.51 | 0.06 |
| Neuro | ETCO₂ | Minimum | 642 | 0.66 | 67.0 | 0.48 | 0.07 |
| Neuro | ETCO₂ | ETCO₂<20 mm Hg | 642 | 0.66 | 67.1 | 0.02 | 0.07 |
| Survival | SpO₂ | Mean | 3,418 | 0.90 | 82.4 | 0.07 | 0.43 |
| Survival | SpO₂ | Minimum | 3,418 | 0.90 | 82.4 | 0.82 | 0.43 |
| Survival | SpO₂ | Min. < 90% | 3,460 | 0.90 | 82.4 | 0.29 | 0.43 |
| Neuro | SpO₂ | Mean | 909 | 0.64 | 67.1 | 0.32 | 0.05 |
| Neuro | SpO₂ | Minimum | 909 | 0.64 | 67.1 | 0.41 | 0.05 |
| Neuro | SpO₂ | Min. < 90% | 917 | 0.64 | 67.5 | 0.69 | 0.05 |
| Survival | RR | Mean | 3,015 | 0.89 | 83.5 | 0.75 | 0.39 |
| Survival | RR | Minimum | 3,015 | 0.89 | 82.3 | 0.69 | 0.38 |
| Survival | RR | RR<8 min | 3,460 | 0.91 | 83.8 | 0.33 | 0.44 |
| Survival | RR | RR>30 min | 3,460 | 0.90 | 82.3 | 0.32 | 0.43 |
| Neuro | RR | Mean | 643 | 0.66 | 67.2 | 0.49 | 0.06 |
| Neuro | RR | Minimum | 643 | 0.66 | 68.0 | 0.16 | 0.06 |
| Neuro | RR | RR<8 min | 917 | 0.64 | 67.1 | 0.07 | 0.05 |
| Neuro | RR | RR>30 min | 917 | 0.64 | 67.4 | 0.28 | 0.05 |

All models adjusted for age, sex, downtime, initial rhythm, witnessed status, arrest location, presumed cardiac aetiology, and resuscitation drugs (adrenaline, amiodarone), with robust standard errors at the patient level. The analytic cohort comprised 3,694 patients with complete survival outcome data; model sample size varied by exposure because physiologic streams or derived measures were unavailable for all patients, and a small number of observations were excluded in some models due to perfect prediction.

*Figure S1 Directed acyclic graph showing hypothesised relationships between post-ROSC physiology (exposure) and survival (outcome), with key confounders*


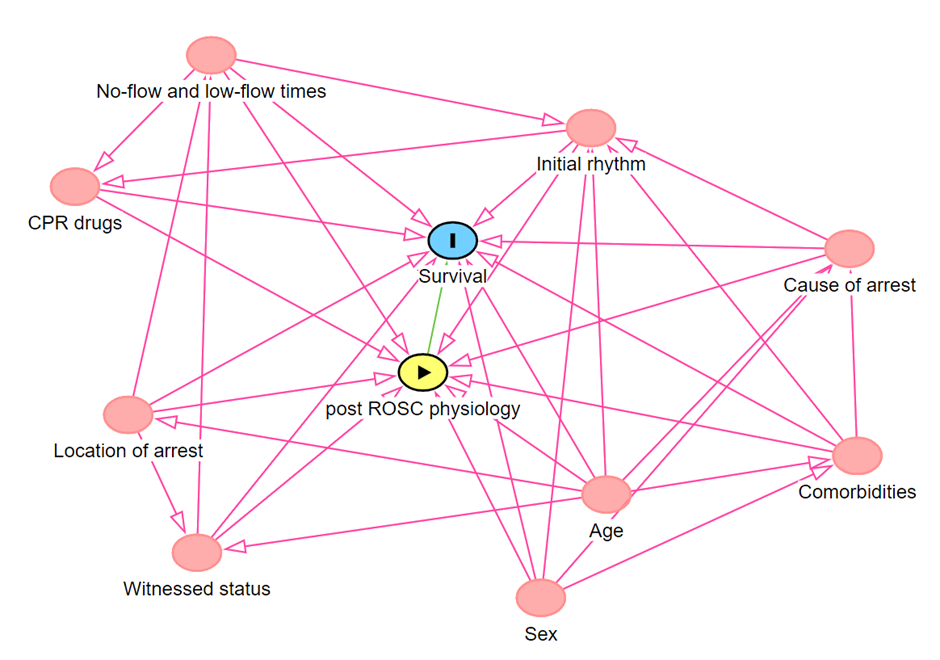


*Figure S2 Predicted survival to hospital discharge by post-ROSC SBP measures: (A) mean SBP, (B) minimum SBP, and (C) minutes with SBP < 90 mm Hg. Shaded areas represent 95% confidence intervals.*


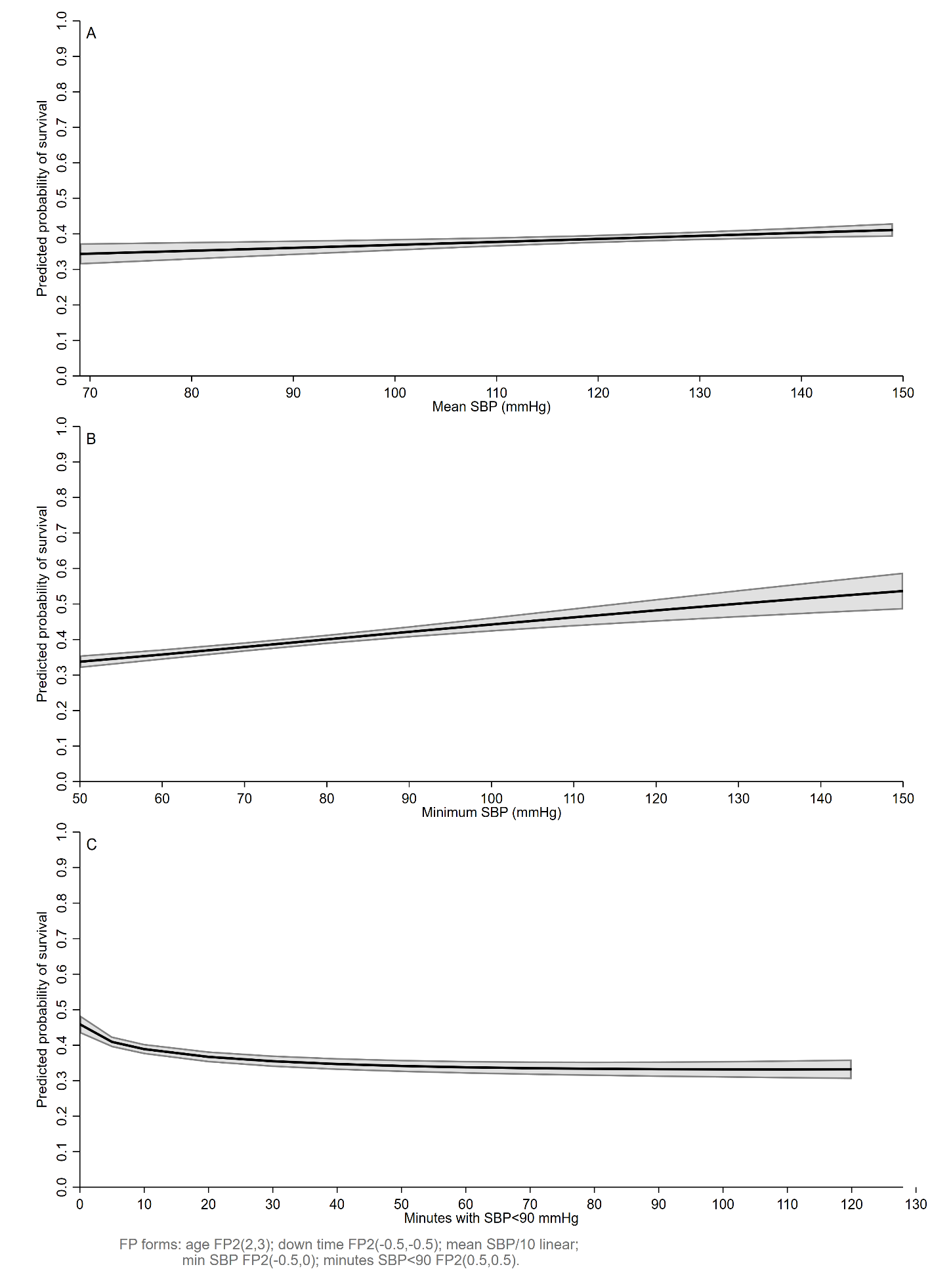


*Figure S3 Predicted probability of good 12-month neurological outcome by post-ROSC SBP measures: (A) mean SBP, (B) minimum SBP, and (C) minutes with SBP < 90 mm Hg. Shaded areas represent 95 % confidence intervals.*


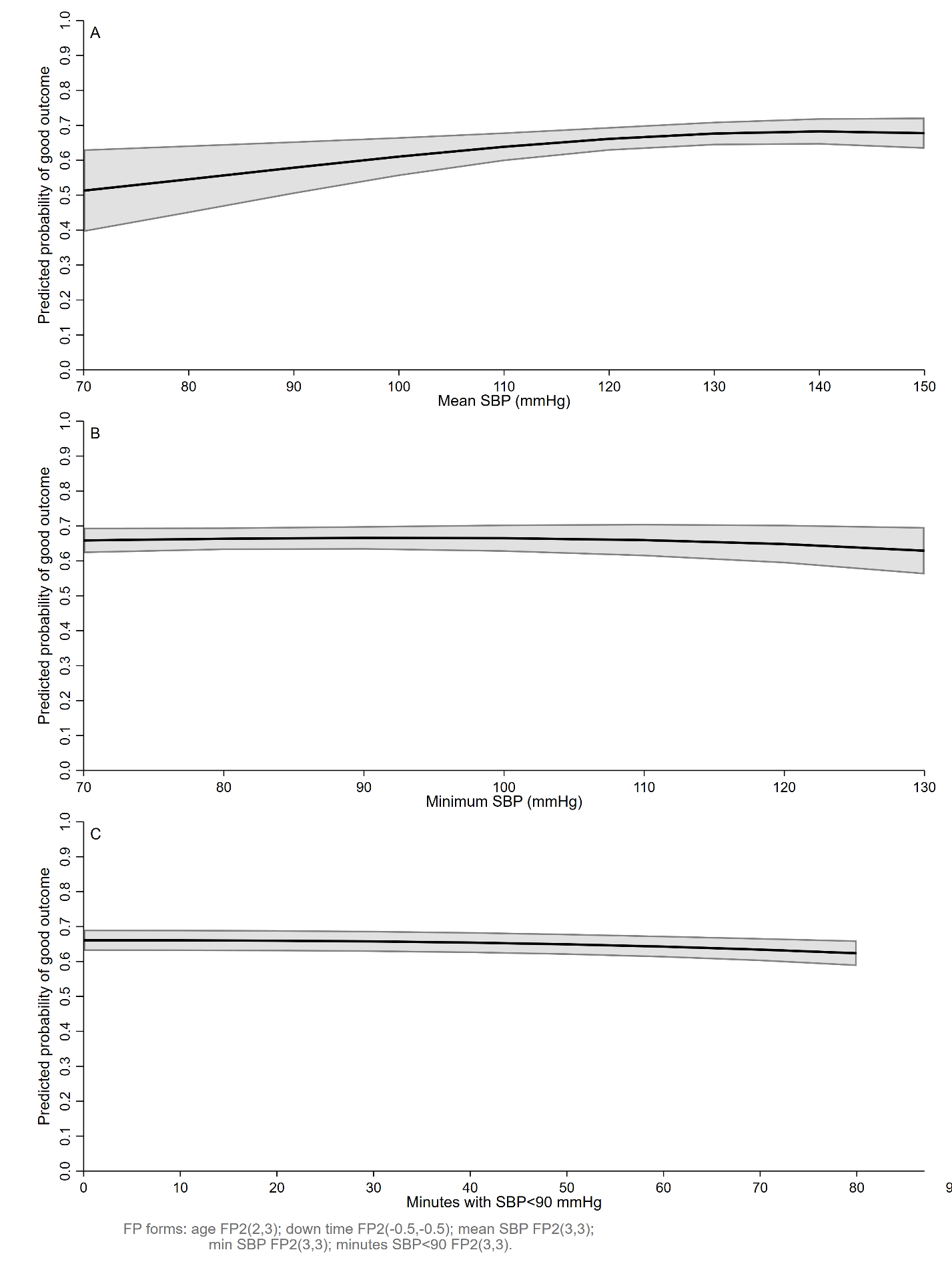


*Figure S4 Predicted survival to hospital discharge by post-ROSC MAP measures: (A) mean MAP, (B) minimum MAP, and (C) minutes with MAP < 65 mm Hg. Shaded areas represent 95% confidence intervals.*


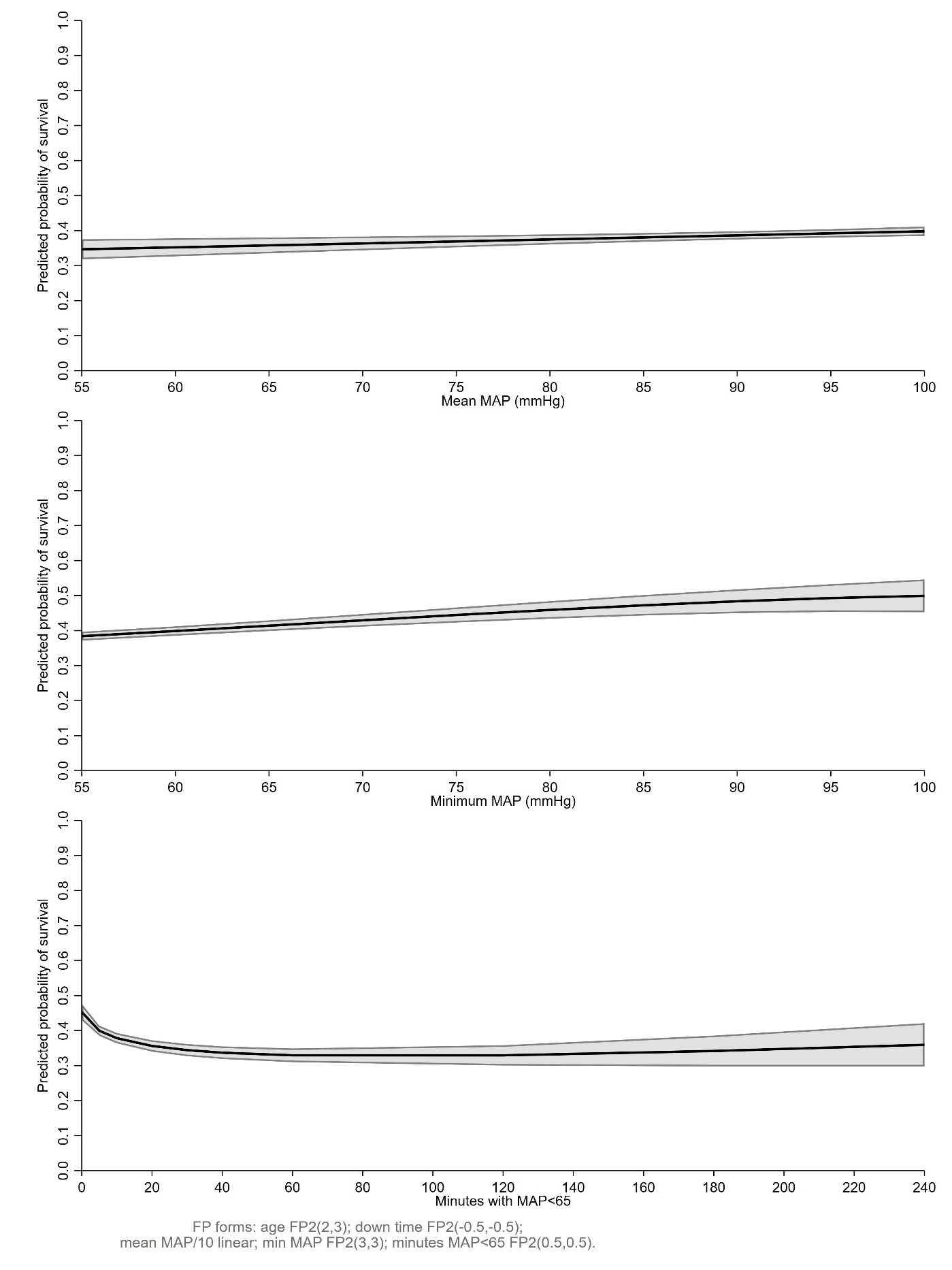


*Figure S5 Predicted probability of good 12-month neurological outcome by post-ROSC MAP measures: (A) mean MAP, (B) minimum MAP, and (C) minutes with MAP < 65 mm Hg. Shaded areas represent 95 % confidence intervals.*


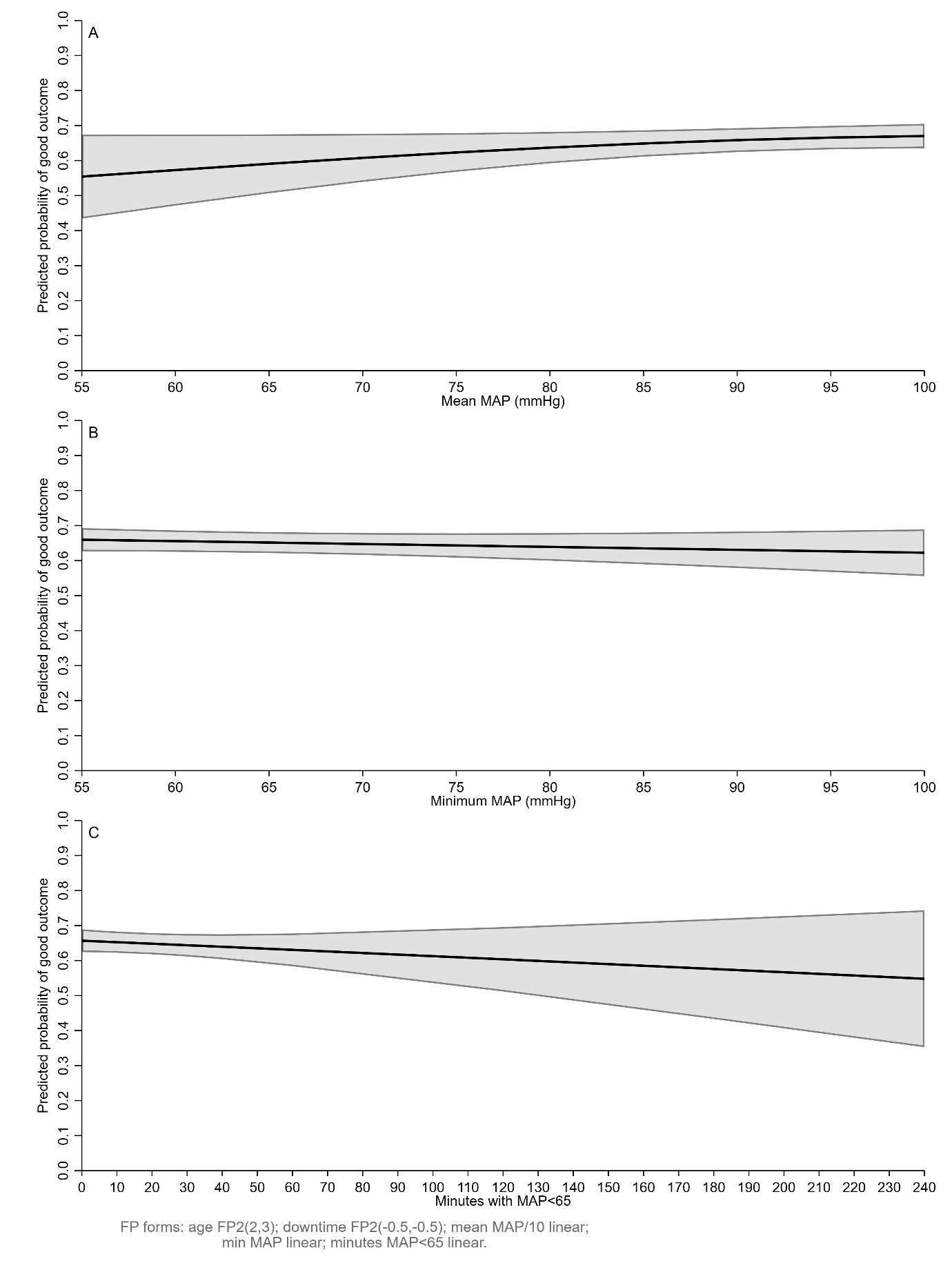


*Figure S6 Predicted survival to hospital discharge by post-ROSC ETCO₂ measures: (A) mean ETCO₂, (B) minimum ETCO₂, and (C) minutes with ETCO₂ < 20 mm Hg. Shaded areas represent 95% confidence intervals.*


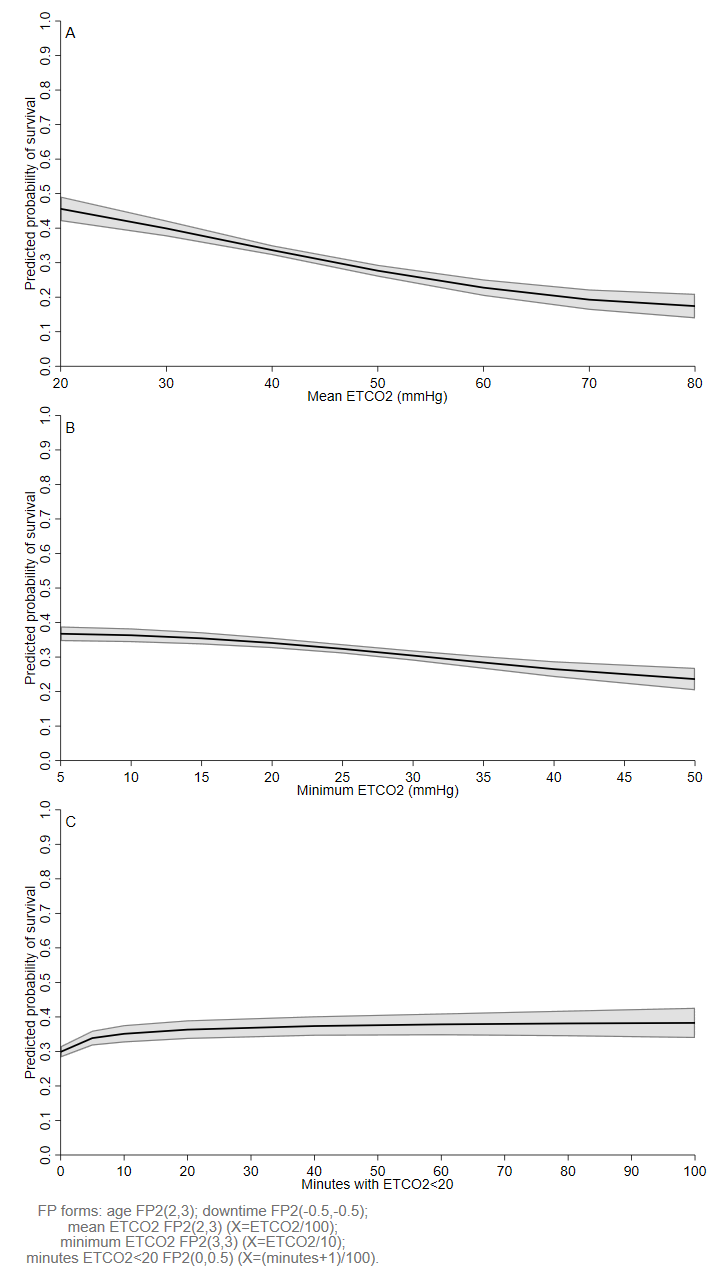


*Figure S7 Predicted probability of good 12-month neurological outcome by post-ROSC ETCO₂ measures: (A) mean ETCO₂, (B) minimum ETCO₂, and (C) minutes with ETCO₂ < 20 mm Hg. Shaded areas represent 95% confidence intervals.*


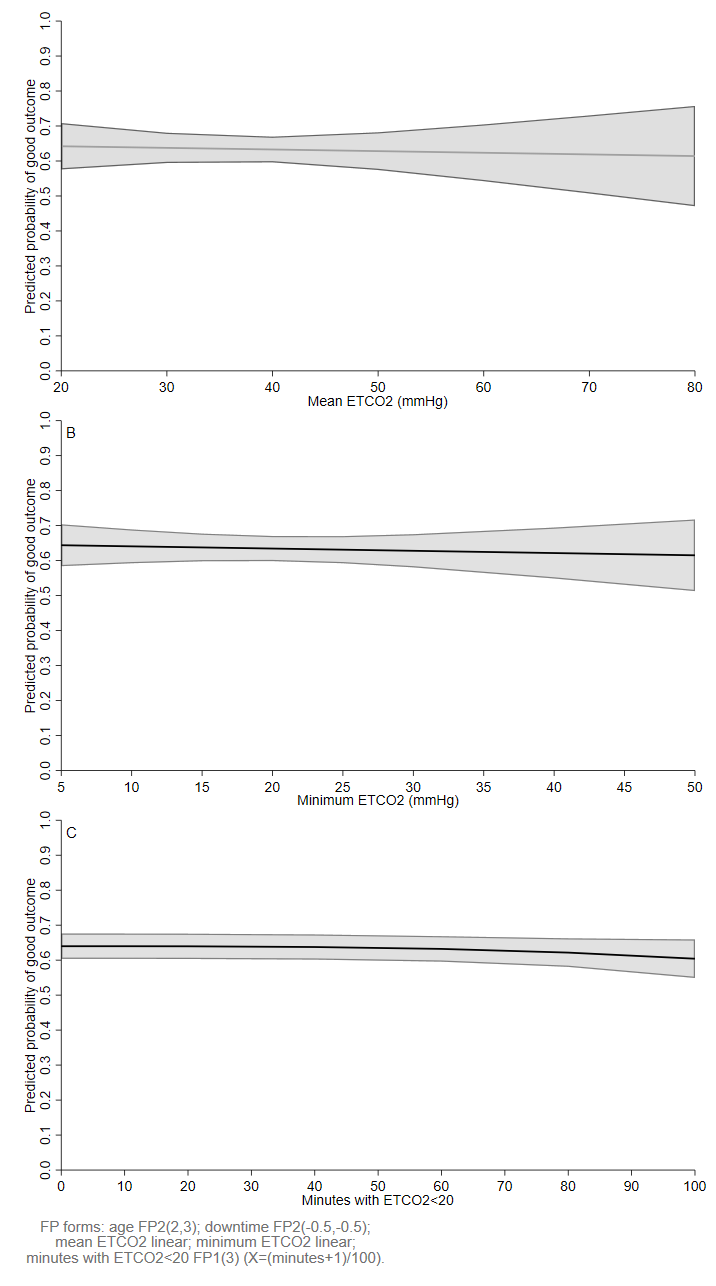


*Figure S8 Predicted survival to hospital discharge by post-ROSC SpO₂ measures: (A) mean SpO₂, (B) minimum SpO₂, and (C) minutes with SpO₂ < 90%. Shaded areas represent 95% confidence intervals.*


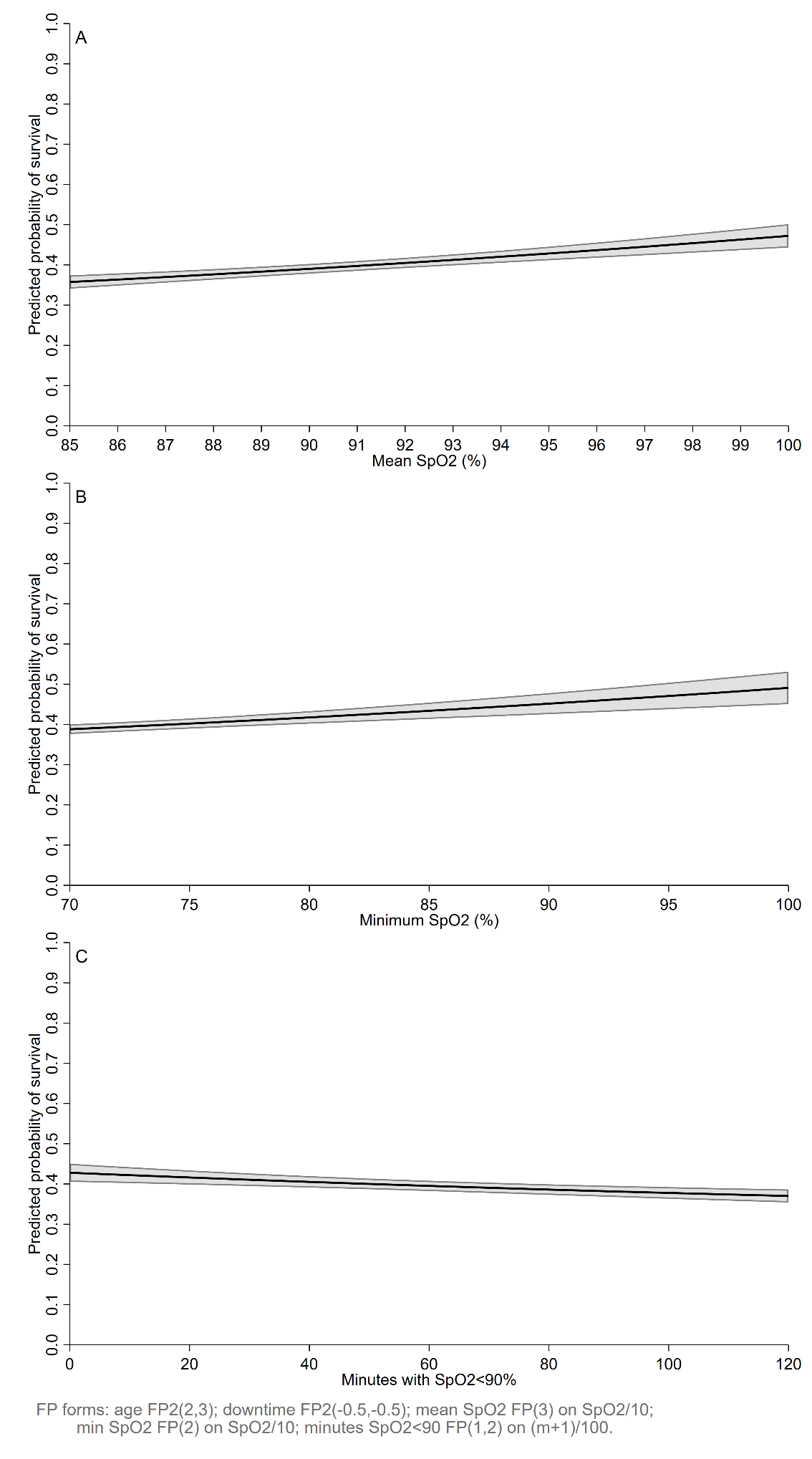


*Figure S9 Predicted probability of good 12-month neurological outcome by post-ROSC SpO₂ measures: (A) mean SpO₂, (B) minimum SpO₂, and (C) minutes with SpO₂ < 90%. Shaded areas represent 95% confidence intervals.*


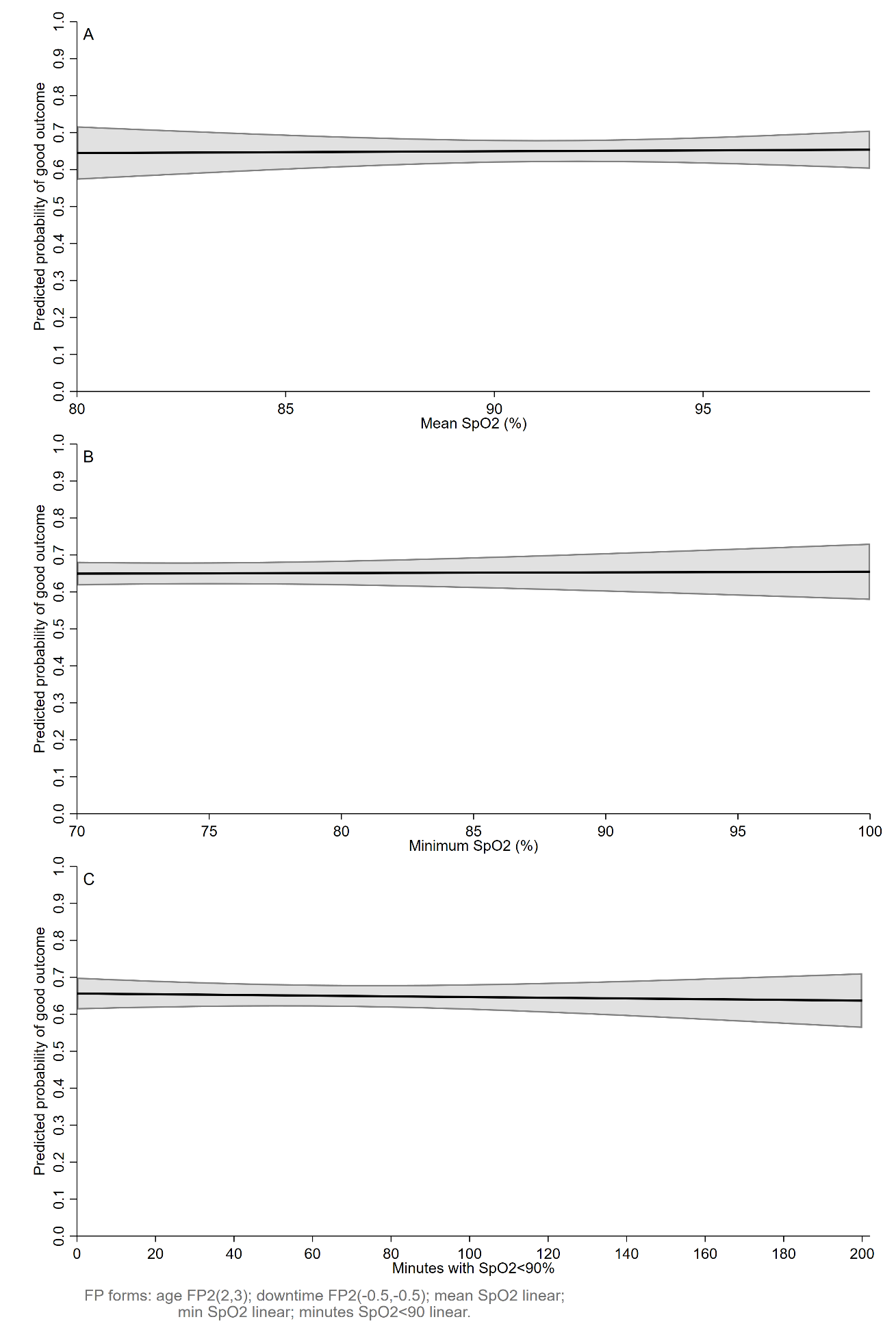


*Figure S10 Predicted survival to hospital discharge by post-ROSC RR measures: (A) mean RR, (B) minimum RR, (C) minutes with RR < 8, and (D) minutes with RR > 30. Shaded areas represent 95% confidence intervals.*


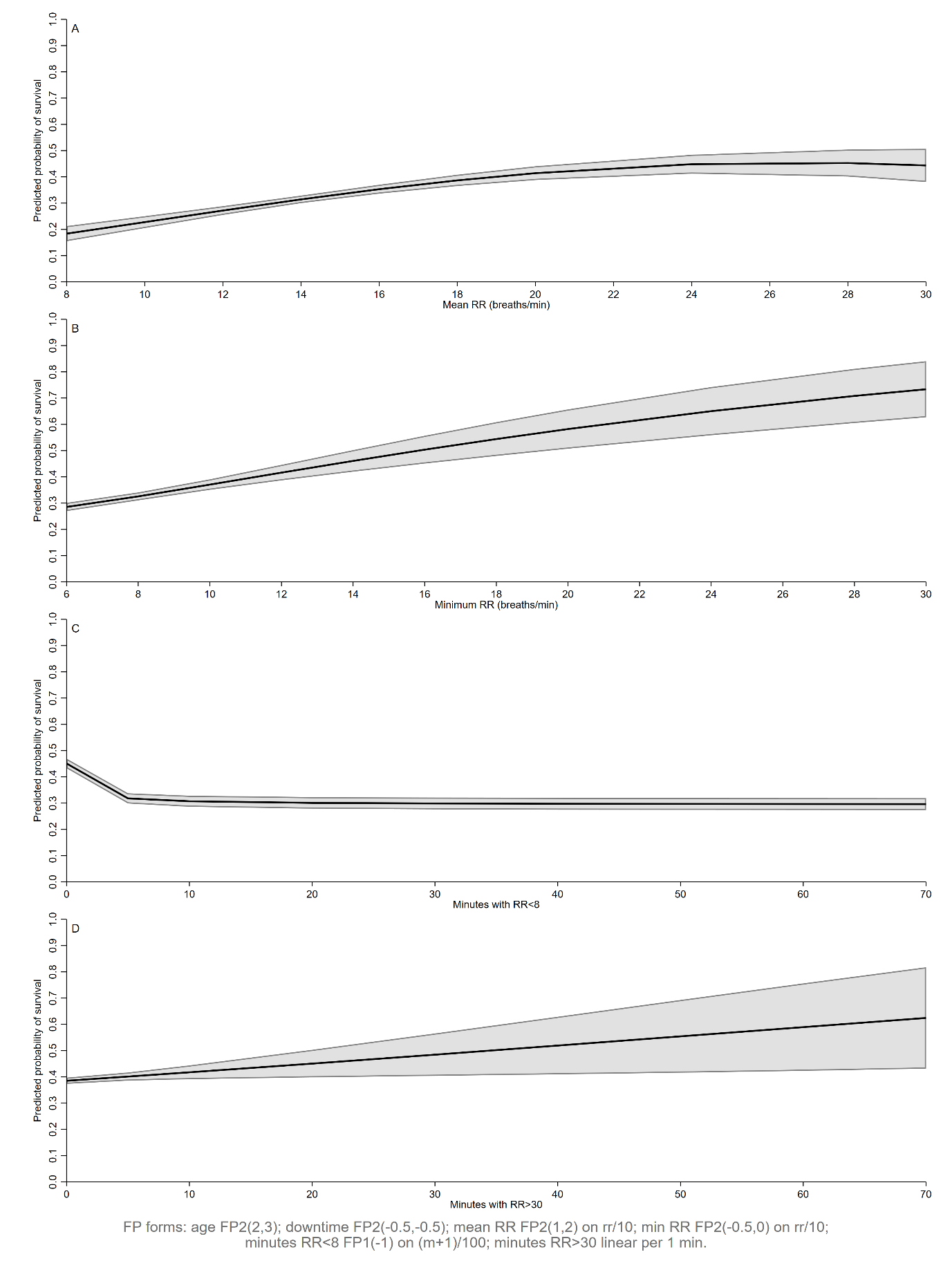


*Figure S11 Predicted probability of good 12-month neurological outcome by post-ROSC RR measures: (A) mean RR, (B) minimum RR, (C) minutes with RR < 8, and (D) minutes with RR > 30. Shaded areas represent 95% confidence intervals.*


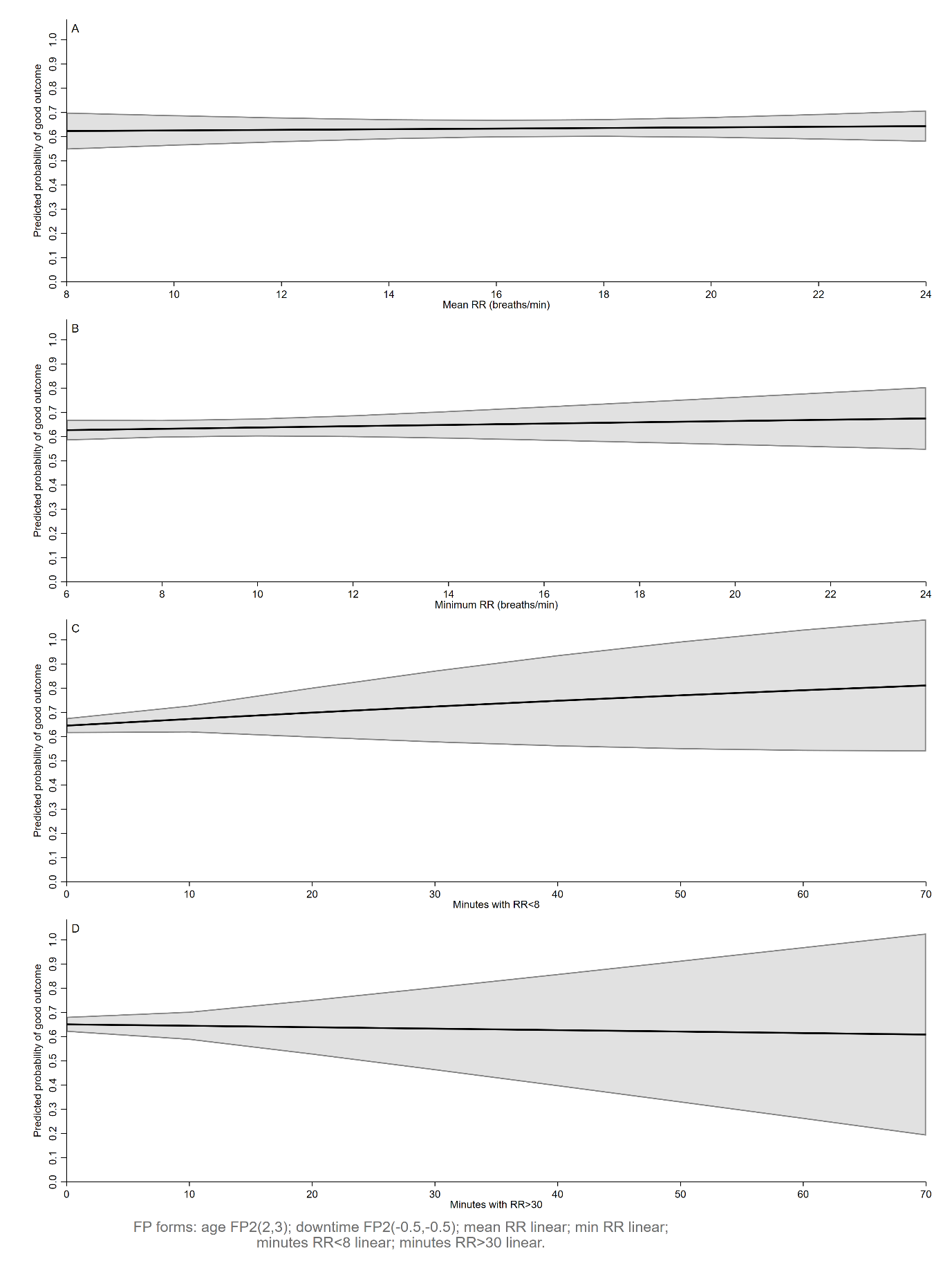


**Supplementary Methods. Monitor-derived physiology quality assurance and data sufficiency**

This section documents monitor signal processing, plausibility screening, missingness patterns, and minimum data requirements for cumulative exposure metrics. It is intended to support interpretability of minute-level vital signs derived from linked monitor-defibrillator files, and to prevent cumulative measures being calculated from sparse data. Quality checks were performed in the first 120 minutes after recorded ROSC, restricting to records with post-ROSC monitor timestamps. Continuous monitoring data were available for 3,779 patients. Median non-missing readings per patient were 242 HR, 159 SpO₂, 125 SBP, 124 MAP, 222 ETCO₂, and 208 RR from the capnography channel. The monitor retained the last valid value until updated, so identical consecutive entries were common. After de-duplication, median distinct readings were 201 HR, 97 SpO₂, 36 blood pressure, 150 ETCO₂, and 103 RR. All checks were conducted without imputing missing values.

**Signal alignment and minute-level aggregation**

Monitor timestamps were aligned to the recorded ROSC time and converted to whole minutes since ROSC. Physiologic streams were aggregated to 1-minute intervals within patient. When multiple readings occurred within a minute, a robust within-minute summary was used, consistent with the primary analysis approach. This reduces sensitivity to transient spikes and avoids over-weighting periods of dense sampling.

**Plausibility screening and handling of artefact**

We applied prespecified plausibility bounds to the raw streams before aggregation and recoded out-of-range values to missing rather than attempting to correct them. These bounds were chosen to exclude values most consistent with probe disconnection, non-measurement states, or clear recording errors, while retaining clinically plausible extremes.

*Table S5 Plausibility bounds and recoding rules applied to raw monitor streams*

| **Stream** | **Lower bound** | **Upper bound** | **Rule** |
| --- | --- | --- | --- |
| HR | 20 | 250 | Keep 20 to 250 bpm |
| SBP | 40 | 300 | Keep 40 to 300 mm Hg |
| DBP | 20 | 200 | Keep 20 to 200 mm Hg |
| MAP | 30 | 200 | Keep 30 to 200 mm Hg |
| SpO2 | 50 | 100 | Keep 50 to 100 percent |
| ETCO2 | 1 | 150 | Keep 1 to 150 mm Hg, non-positive set missing |
| RR | 2 | 100 | Keep 2 to 100 breaths/min |

Values outside bounds were set to missing prior to minute-level aggregation. ETCO2 values at or below zero were treated as a non-measurement state and recoded as missing.

*Table S6 Frequency of values removed by plausibility screening*

| **Stream** | **Raw N** | **After screening N** | **Removed N** | **Removed, %** |
| --- | --- | --- | --- | --- |
| HR | 709,333 | 708,290 | 1,043 | 0.15 |
| SBP | 437,435 | 436,330 | 1,105 | 0.25 |
| DBP | 436,893 | 435,941 | 952 | 0.22 |
| MAP | 435,404 | 434,193 | 1,211 | 0.28 |
| SpO2 | 524,625 | 516,152 | 8,473 | 1.62 |
| ETCO2 | 569,644 | 569,644 | 0 | 0.00 |
| RR | 525,058 | 525,058 | 0 | 0.00 |

Plausibility screening removed a small proportion of values across streams, with the largest removal proportion observed for SpO2. This pattern is consistent with intermittent poor plethysmography signal and brief probe dropouts in the early post-arrest period.

**Distributional checks and extremes**

We report raw and cleaned reading-level percentiles to show the observed ranges and tails of each stream, followed by the corresponding minute-level percentiles after aggregation. The minute-level distributions were clinically plausible and consistent with a mixed post-ROSC cohort.

*Table S7 Reading-level distributions and extremes for post-ROSC vital signs (raw vs after plausibility screening)*

| **Stage** | **Stream** | **N** | **Min** | **P1** | **Median** | **P99** | **Max** |
| --- | --- | --- | --- | --- | --- | --- | --- |
| Raw | HR (bpm) | 709,333 | 20 | 37 | 102 | 197 | 300 |
| Cleaned | HR (bpm) | 708,290 | 20 | 37 | 102 | 194 | 250 |
| Raw | SBP (mm Hg) | 437,435 | 10 | 52 | 127 | 219 | 260 |
| Cleaned | SBP (mm Hg) | 436,330 | 40 | 55 | 127 | 219 | 260 |
| Raw | MAP (mm Hg) | 435,404 | 7 | 40 | 95 | 165 | 228 |
| Cleaned | MAP (mm Hg) | 434,193 | 30 | 41 | 95 | 164 | 200 |
| Raw | SpO2 (%) | 524,625 | 1 | 41 | 95 | 100 | 100 |
| Cleaned | SpO2 (%) | 516,152 | 50 | 58 | 95 | 100 | 100 |
| Raw | ETCO2 (mm Hg) | 569,644 | 2 | 11 | 42 | 115 | 149 |
| Cleaned | ETCO2 (mm Hg) | 569,644 | 2 | 11 | 42 | 115 | 149 |
| Raw | RR (breaths/min) | 525,058 | 2 | 5 | 14 | 36 | 100 |
| Cleaned | RR (breaths/min) | 525,058 | 2 | 5 | 14 | 36 | 100 |

Percentiles are calculated across all recorded readings in the 0-to-120-minute window. Screened values reflect recoding of out-of-range readings to missing. These checks support that outliers were rare and that central tendencies were stable before and after screening.

*Table S8 Minute-level distributions and extremes for post-ROSC vital signs (after plausibility screening and aggregation)*

| **Stream** | **N (minute values)** | **Min** | **P1** | **Median** | **P99** | **Max** |
| --- | --- | --- | --- | --- | --- | --- |
| SBP (mm Hg) | 194,686 | 40 | 54 | 125 | 218 | 260 |
| MAP (mm Hg) | 193,599 | 30 | 40 | 94 | 163 | 200 |
| SpO2 (%) | 197,844 | 50 | 58 | 95 | 100 | 100 |
| ETCO2 (mm Hg) | 209,738 | 2 | 11.5 | 41.7 | 113 | 149 |
| RR (breaths/min) | 205,786 | 2 | 5 | 13.5 | 34 | 100 |
| HR (bpm) | 253,593 | 20 | 38 | 101.5 | 187 | 250 |

Minute-level percentiles reflect one aggregated value per patient-minute. For SpO2, values show expected upper-bound clustering at 100%, consistent with saturation ceiling effects in a setting where high inspired oxygen is common. For blood pressure, ranges are consistent with post-ROSC haemodynamic recovery and intermittent NIBP measurement.

*Figure S12 Minute-level distributions of SBP, MAP, SpO2, and ETCO2 (0 to 120 minutes)*

| 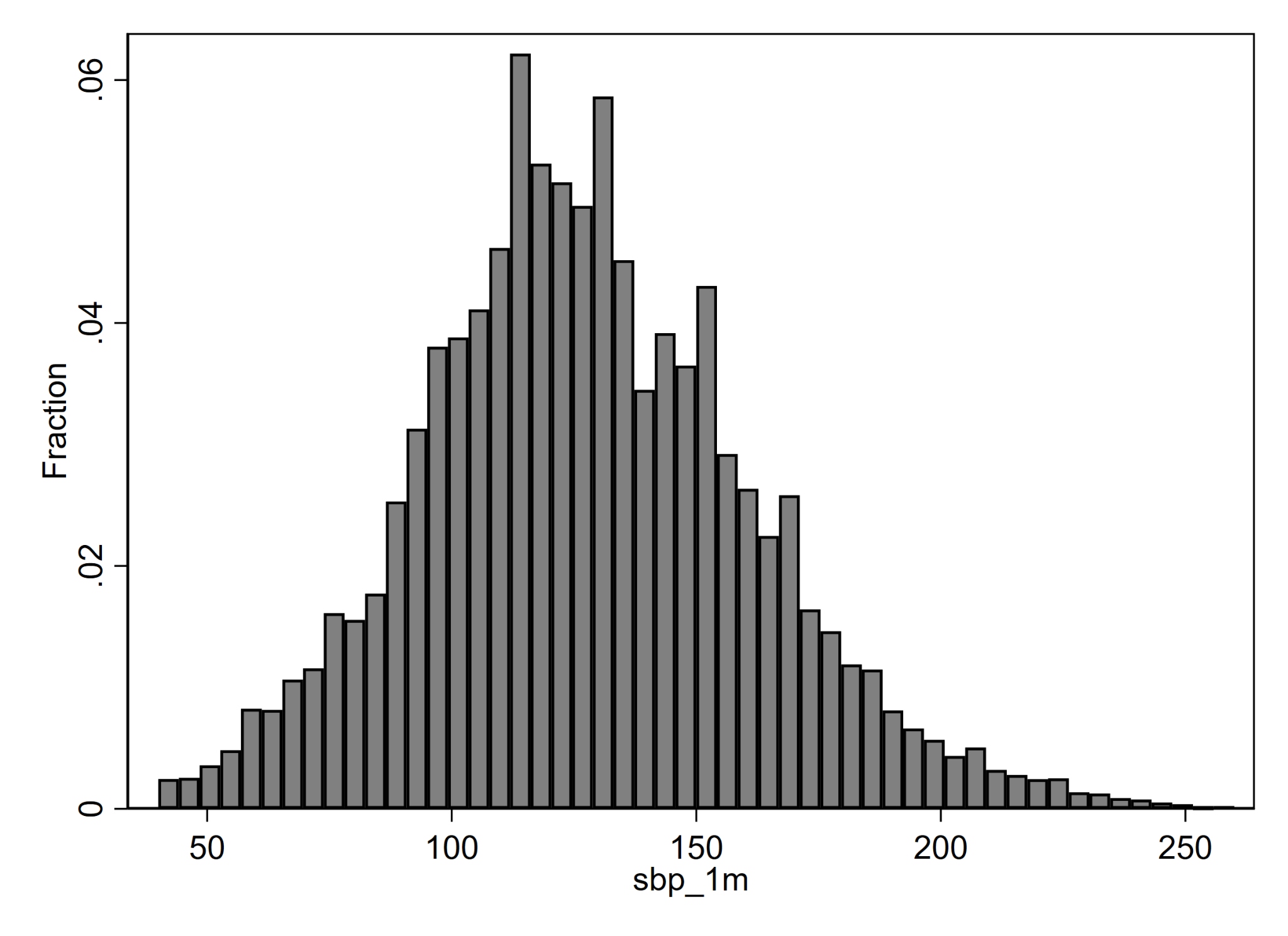 | 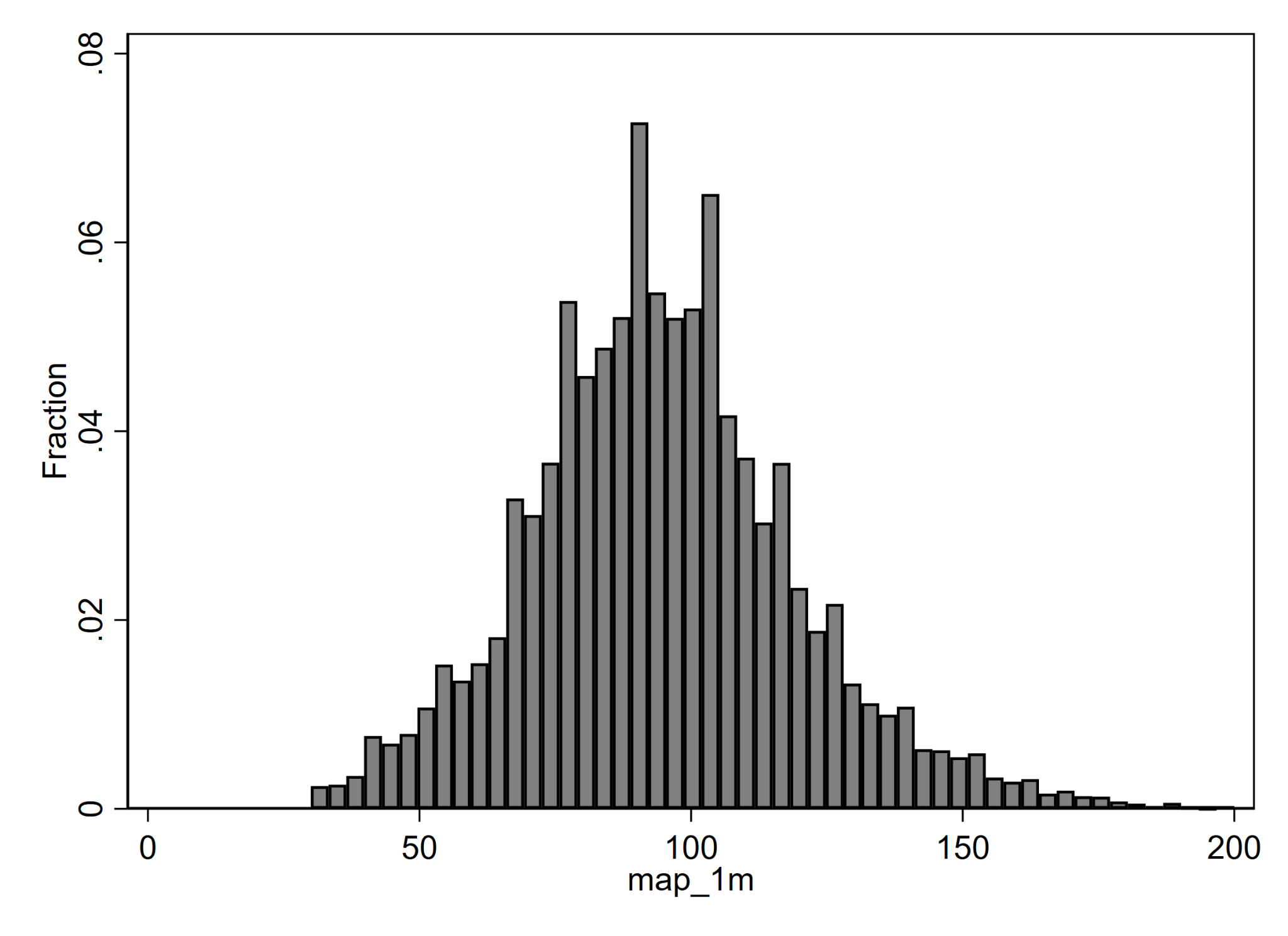 |
| --- | --- |
| 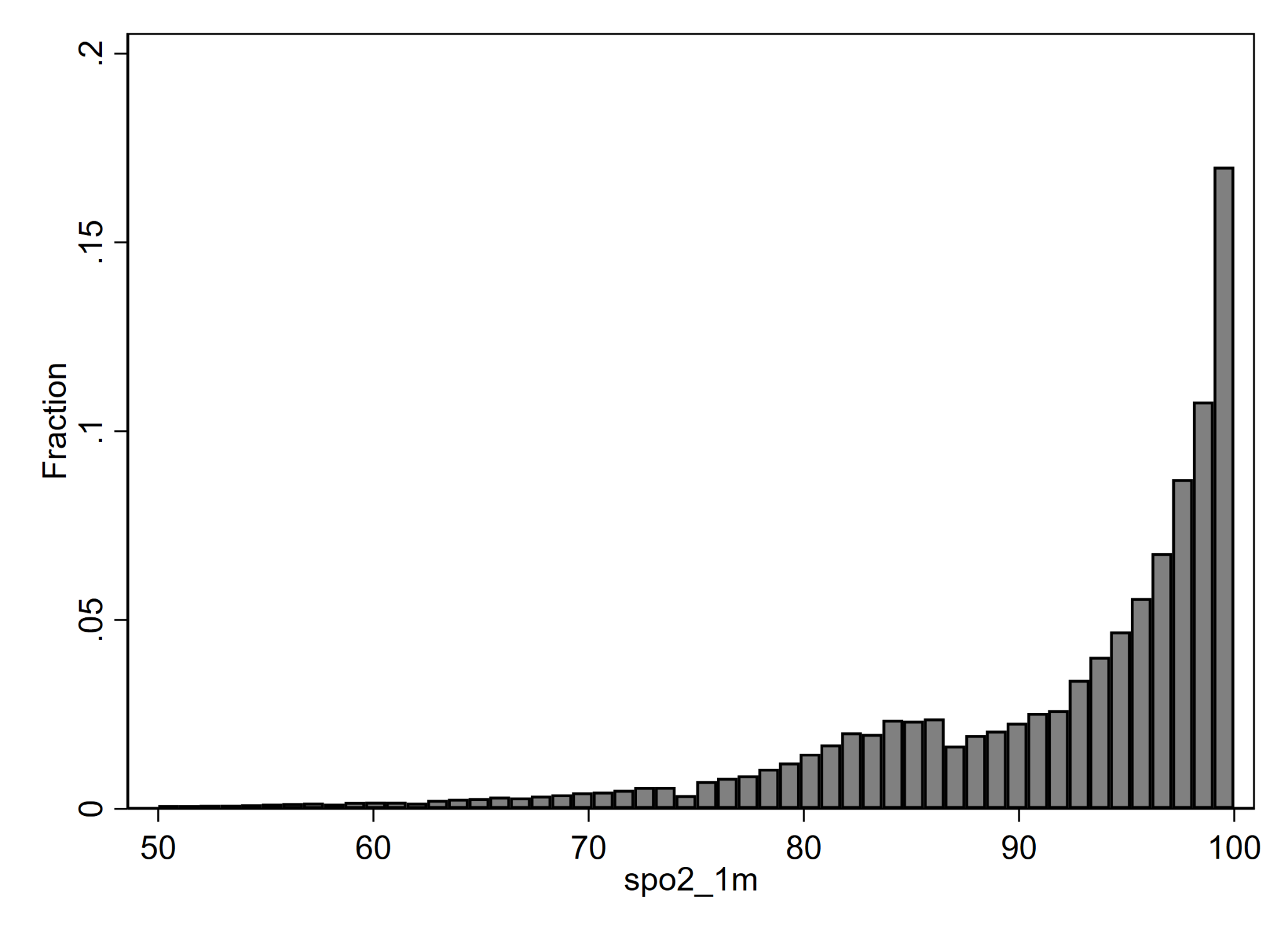 | 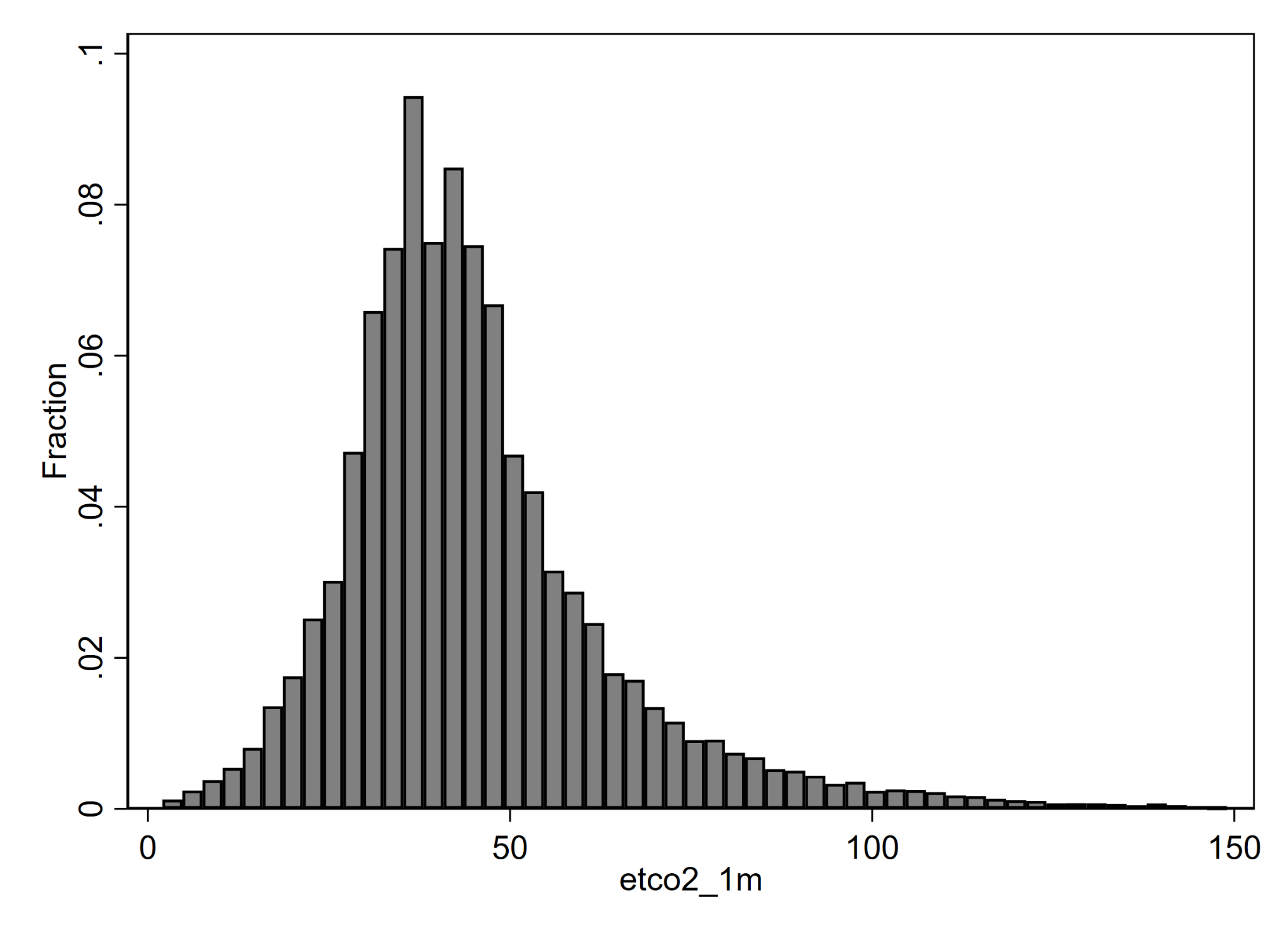 |

Histograms show one value per patient-minute after plausibility screening and minute-level aggregation.

*Figure S13 Minute-level distribution of respiratory rate (RR, 0 to 120 minutes)*


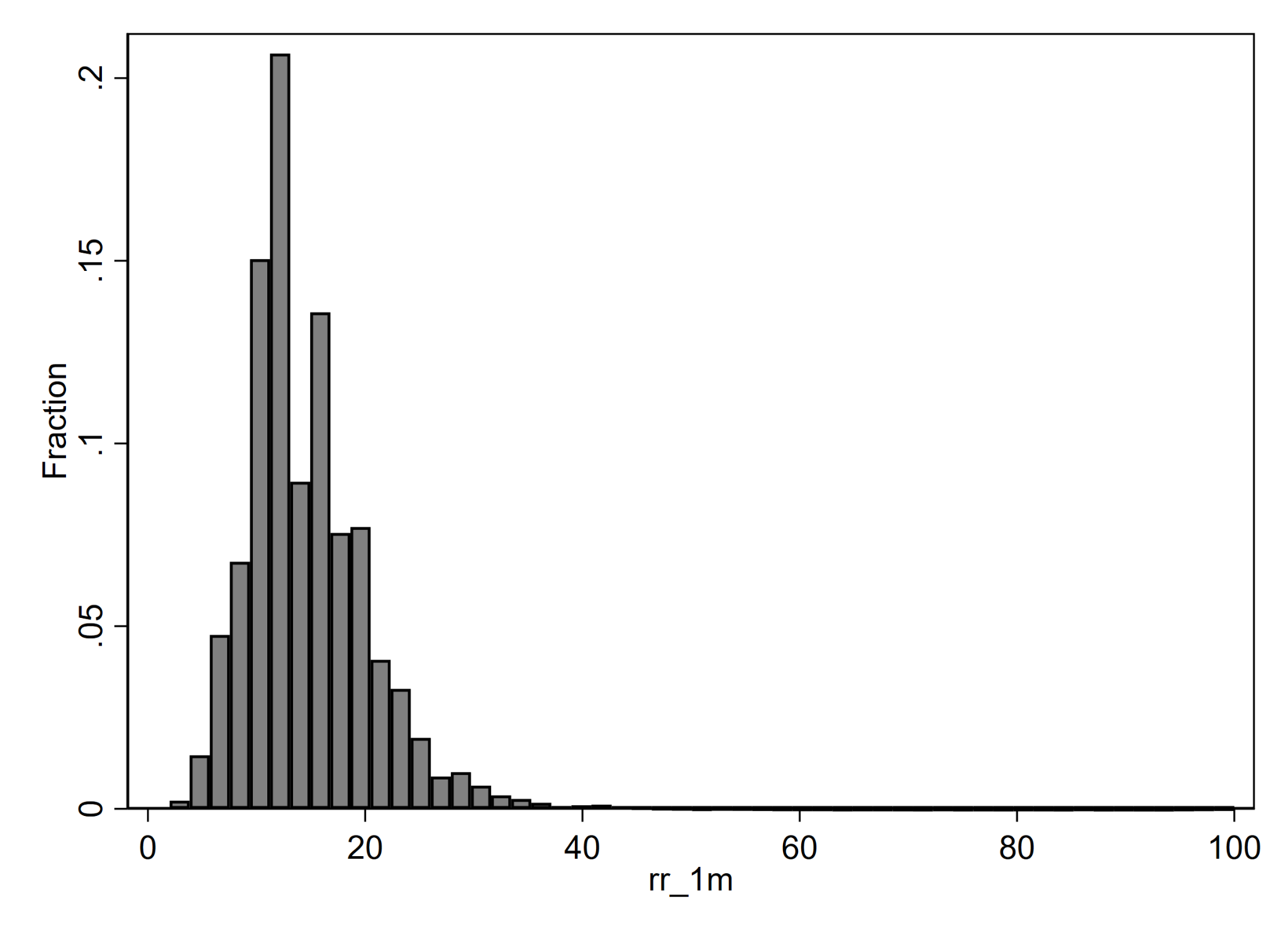


RR is derived from the capnography channel where available. The distribution is consistent with a mix of spontaneous breathing and clinician-delivered ventilation.

**Missingness and monitoring availability over time**

Missingness was quantified as the proportion of patient-minutes without an available minute-level value at each minute since ROSC. This provides a transparent description of monitoring availability and highlights streams that are inherently intermittent (NIBP) or prone to signal loss (SpO2), as well as truncation of monitoring as prehospital care ends.

*Table S9 Monitoring density per patient in the first 120 minutes after ROSC*

| **Stream** | **Raw datapoints per patient, median (IQR)** | **Monitored minutes per patient, median (IQR)** | **Distinct minute-level values, median (IQR)** |
| --- | --- | --- | --- |
| SBP | 117 (81 to 154) | 53 (38 to 68) | 17 (9 to 26) |
| MAP | 116 (81 to 153) | 52 (37 to 68) | 17 (9 to 25) |
| SpO2 | 143 (92 to 191) | 54 (36 to 71) | 42 (27 to 58) |
| ETCO2 | 169 (105 to 215) | 63 (39 to 79) | 57 (37 to 71) |
| RR | 157 (94 to 200) | 62 (38 to 77) | 34 (21 to 48) |
| HR | 191 (147 to 240) | 68 (54 to 85) | 61 (48 to 76) |

Raw datapoints count all recorded readings. Monitored minutes count the number of minutes with at least one valid minute-level value. Distinct minute-level values are calculated after removing consecutive duplicates and are most informative for NIBP measures.

*Table S10 NIBP update interval summary (minutes between distinct SBP or MAP values)*

| **Metric** | **Distinct updates (N)** | **P25** | **Median** | **P75** | **P90** | **P95** |
| --- | --- | --- | --- | --- | --- | --- |
| SBP | 63,653 | 2 | 2 | 4 | 6 | 9 |
| MAP | 62,632 | 2 | 2 | 4 | 6 | 9 |

Intervals are computed from the minute-level series after removing consecutive duplicates. These summaries support that NIBP values update intermittently, and that long runs of identical values are consistent with carry-forward display behaviour rather than repeated new cuff measurements.

*Table S11 Proportion missing at selected minutes since ROSC (minute-level series)*

| **Minutes since ROSC** | **SBP missing, %** | **MAP missing, %** | **SpO2 missing, %** | **ETCO2 missing, %** | **RR missing, %** |
| --- | --- | --- | --- | --- | --- |
| 0 | 81.6 | 81.8 | 62.1 | 33.1 | 37.0 |
| 5 | 29.1 | 29.3 | 41.7 | 26.1 | 28.9 |
| 10 | 27.9 | 28.2 | 34.3 | 24.5 | 26.6 |
| 20 | 26.1 | 26.3 | 28.7 | 24.1 | 25.9 |
| 30 | 25.9 | 26.6 | 26.2 | 24.9 | 26.1 |
| 60 | 48.2 | 48.4 | 45.7 | 43.2 | 43.6 |
| 90 | 83.9 | 84.0 | 81.8 | 81.9 | 82.0 |
| 120 | 94.8 | 94.8 | 94.5 | 95.0 | 95.1 |

At minute 0, missingness is highest for NIBP and SpO2, consistent with delay to first cuff measurement and stabilisation of plethysmographic signal. Missingness is lowest around 20 to 30 minutes and then rises as monitoring ends, consistent with truncation at the end of prehospital care or during handover.

*Figure S14 Missingness over time for minute-level post-ROSC physiology (0 to 120 minutes)*


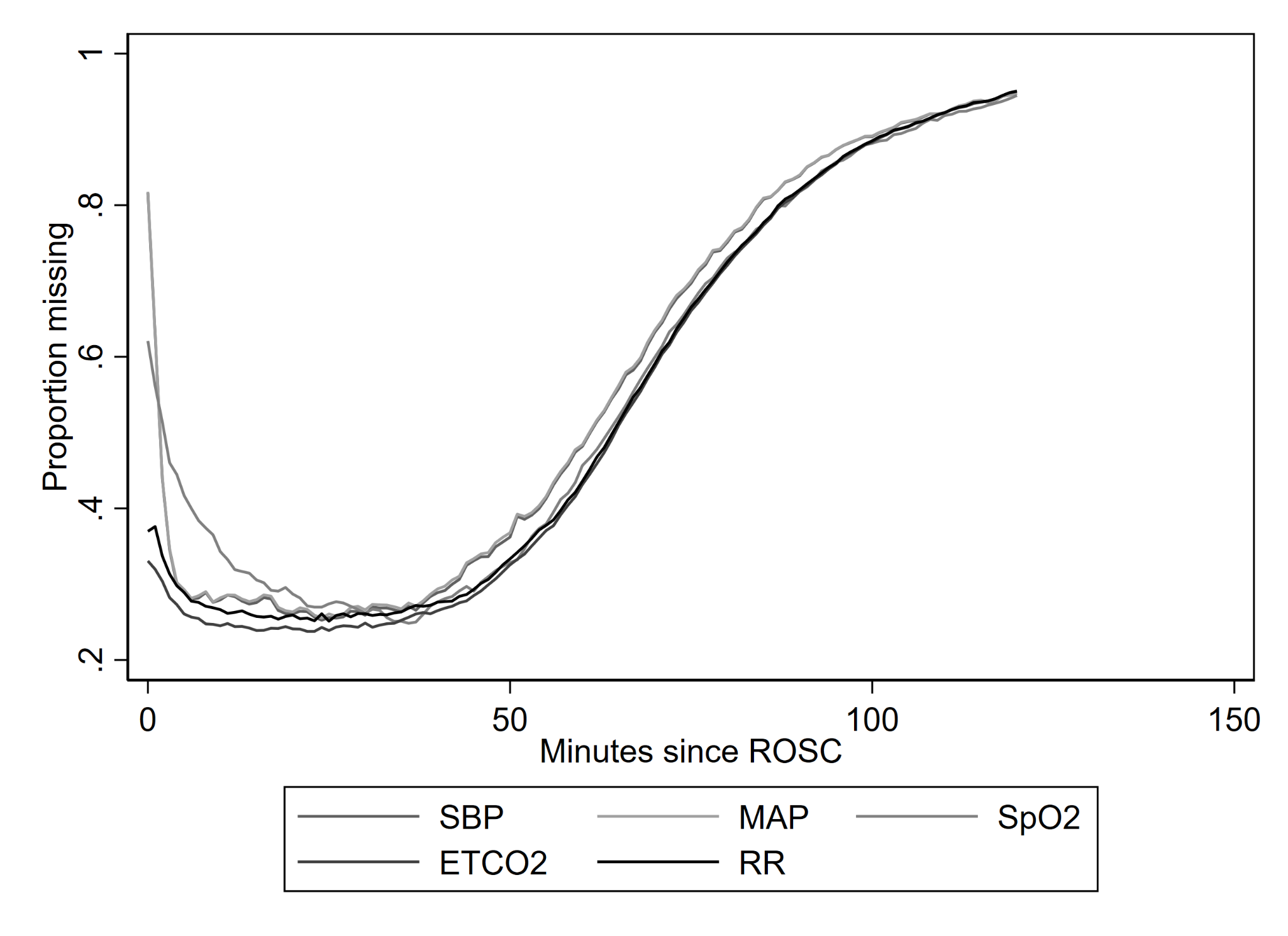


Curves represent the proportion of patients missing each stream at each minute since ROSC. The pattern is consistent with early stabilisation and later truncation of monitoring rather than random missingness.

**Minimum data requirements for cumulative exposure metrics**

To prevent cumulative exposure measures such as minutes below threshold or area under threshold being calculated from sparse data, we prespecified minimum data requirements. For SpO2, ETCO2 , and RR cumulative metrics we required at least 10 monitored minutes in the analysis window. For SBP and MAP cumulative metrics we required at least 10 monitored minutes and at least 3 distinct values after removing consecutive duplicates. Patients not meeting these criteria were coded as missing for the relevant cumulative metric.

*Table S12 Eligibility for cumulative exposure metrics under minimum data requirements*

| **Metric** | **Patients assessed** | **Eligible for cumulative metrics** | **Eligible, %** |
| --- | --- | --- | --- |
| SBP | 3,659 | 3,499 | 95.6 |
| MAP | 3,659 | 3,489 | 95.4 |
| SpO2 | 3,659 | 3,472 | 94.9 |
| ETCO2 | 3,659 | 3,095 | 84.6 |
| RR | 3,659 | 3,093 | 84.5 |

Among patients not meeting SBP or MAP criteria, the typical monitoring volume was low (median 6 to 7 monitored minutes) with only 1 distinct NIBP value, consistent with one or two cuff cycles. For SpO2, patients failing criteria typically had very few monitored minutes (median 2). For ETCO2 and RR, ineligible patients typically had no capnography-derived values in the window (median 0 monitored minutes), consistent with absence of capnography rather than intermittent signal loss.

**Interpretation notes for capnography-derived measures and oxygen saturation**

Respiratory rate and ETCO2 were obtained from the capnography channel. These measures are influenced by clinical context and ventilation strategy, and in ventilated patients RR often reflects delivered ventilation rather than intrinsic respiratory drive. Because airway management and ventilation mode can vary during the post-ROSC period, and airway interventions may be sequential within a single episode, we did not attempt to stratify or adjust exposure distributions by airway type or ventilatory mode in these supplementary QC analyses. Accordingly, ETCO2 and RR results in the main analyses are interpreted as context-dependent associations among cases with capnography available, rather than as universal physiologic targets. For oxygen saturation, prehospital management commonly involves high inspired oxygen. In that setting, SpO2 is best interpreted as a marker of oxygenation failure and severity when low, while high SpO2 does not imply hyperoxaemia and cannot distinguish degrees of hyperoxia because of saturation plateauing. Therefore, the main oxygenation analyses focus on hypoxaemia and cumulative time below threshold rather than inferring oxygen dose.
